# AMPK-dependent phosphorylation of MTFR1L regulates mitochondrial morphology

**DOI:** 10.1126/sciadv.abo7956

**Published:** 2022-11-11

**Authors:** Lisa Tilokani, Fiona M. Russell, Stevie Hamilton, Daniel M. Virga, Mayuko Segawa, Vincent Paupe, Anja V. Gruszczyk, Margherita Protasoni, Luis-Carlos Tabara, Mark Johnson, Hanish Anand, Michael P. Murphy, D. Grahame Hardie, Franck Polleux, Julien Prudent

**Affiliations:** ^1^ Medical Research Council Mitochondrial Biology Unit, University of Cambridge, Hills Road, CB2 0XY Cambridge, UK.; ^2^ Division of Cell Signalling & Immunology, School of Life Sciences, University of Dundee, Dundee DD1 5EH, Scotland, UK.; ^3^ Department of Neuroscience, Columbia University, New York, NY 10032, USA.; ^4^ Department of Medicine, University of Cambridge, Cambridge, UK.

## Abstract

Mitochondria are dynamic organelles that undergo membrane remodeling events in response to metabolic alterations to generate an adequate mitochondrial network. Here, we investigated the function of mitochondrial fission regulator 1-like protein (MTFR1L), an uncharacterized protein that has been identified in phosphoproteomic screens as a potential AMP-activated protein kinase (AMPK) substrate. We showed that MTFR1L is an outer mitochondrial membrane–localized protein modulating mitochondrial morphology. Loss of MTFR1L led to mitochondrial elongation associated with increased mitochondrial fusion events and levels of the mitochondrial fusion protein, optic atrophy 1. Mechanistically, we show that MTFR1L is phosphorylated by AMPK, which thereby controls the function of MTFR1L in regulating mitochondrial morphology both in mammalian cell lines and in murine cortical neurons in vivo. Furthermore, we demonstrate that MTFR1L is required for stress-induced AMPK-dependent mitochondrial fragmentation. Together, these findings identify MTFR1L as a critical mitochondrial protein transducing AMPK-dependent metabolic changes through regulation of mitochondrial dynamics.

## Teaser

Functional characterization of MTFR1L as a mitochondrial AMPK substrate controlling mitochondrial dynamics.

## INTRODUCTION

Mitochondria form a dynamic and interconnected network, which is constantly remodeled by cycles of membrane fission and fusion to ensure adequate shape, size, and cellular distribution (*1*). This dynamic regulation of mitochondrial structure underlies remarkable functional adaptations of this unique organelle: in general, mitochondria adopting a highly tubular and connected network display high oxidative phosphorylation (OXPHOS), whereas fragmented and small mitochondria tend to be associated with lower OXPHOS capacity and low adenosine triphosphate (ATP) production (*2*). Mitochondrial fission, or division, encompasses a complex multistep series of events mediated by cooperation between core members of the mitochondrial fission machinery and that of other organelles (*3*–*5*). The large guanosine triphosphatase (GTPase) dynamin-related protein-1, Drp1, the principal player in mitochondrial division, is recruited to mitochondrial constriction sites, which are induced by close association with the endoplasmic reticulum (ER) (*6*), through its outer mitochondrial membrane (OMM) receptors such as mitochondrial fission factor (MFF) (*7*) and/or mitochondrial dynamics protein of 49 kDa (MiD49) (*8*) and protein of 51 kDa (MiD51) (*9*). At these mitochondrial constriction sites, Drp1 oligomerizes, constricting the membrane and inducing mitochondrial division in a guanosine triphosphate (GTP)–dependent manner (*10*–*12*), with the contribution of lysosomes (*13*) and trans-Golgi network vesicles (*14*). In contrast, mitochondrial fusion is a coordinated two-step mechanism executed sequentially by OMM fusion, followed by inner mitochondrial membrane (IMM) fusion (*15*, *16*). OMM fusion is mainly mediated by homo- or heterotypic interactions of the large GTPases mitofusin 1 and 2 (Mfn1/2) (*17*), which upon GTP hydrolysis and conformational change drive membrane fusion (*18*). This event is followed by fusion of the IMM, which is regulated by the heterotypic interaction between the large GTPase optic atrophy 1 (OPA1) and cardiolipin at the contact site of opposing membranes (*19*). An imbalance between these two opposing processes of mitochondrial division and fusion has been reported across multiple disorders from cancer to neurodegeneration (*20*), which highlights their physiological relevance and the need to elucidate their mechanisms of regulation.

These dynamic shape transitions of the mitochondrial network represent a crucial response to cellular needs and are adaptations to energetic stress (*2*). While these two antagonistic processes are spatiotemporally coordinated and regulated (*21*–*23*), the underlying signaling pathways that regulate these events have not been fully elucidated. Adenosine 5′-monophosphate (AMP)–activated protein kinase (AMPK), an essential kinase that senses cellular metabolites, is activated upon decreases in cellular energy status or nutrient availability, initiating cascades of phosphorylation reactions that act to restore energy homeostasis, by inhibiting anabolism and promoting catabolic processes (*24*). With an array of substrates involved in different functions, AMPK has been shown to localize to different subcellular compartments, including to the surface of the OMM (*25*–*27*). Increasing numbers of mitochondrial substrates for AMPK, involved in stimulation of mitochondrial biogenesis, regulation of mitochondrial quality control and motility, calcium homeostasis, and dynamics, have now been identified (*28*). These findings highlight AMPK as a crucial regulator of mitochondrial homeostasis. For example, during mitochondrial stress induced by electron transport chain (ETC) dysfunction and a decline in ATP production, activated AMPK phosphorylates MFF, leading to enhanced recruitment of Drp1 to mitochondria (*29*). This leads to mitochondrial fragmentation (*29*), which is thought to increase mitochondrial turnover through mitophagy and allow increased mitochondrial biogenesis (*28*). In addition, Armadillo repeat containing protein 10 (ARMC10) has recently been shown to be an additional substrate of AMPK involved in the regulation of mitochondrial morphology (*30*), revealing the critical and complex role of AMPK signaling in the regulation of mitochondrial dynamics.

Recent high-throughput phosphoproteomic screens have identified potential AMPK substrates including an uncharacterized protein of unknown function, called mitochondrial fission regulator 1-like (MTFR1L) or FAM54B (*25*, *30*, *31*). In these studies, two potential AMPK phosphorylation sites at conserved residues Ser^103^ (*31*) and Ser^238^ (*30*) have been identified. However, their validation and characterization of their functional relevance have not been performed. Phylogenetic analysis of protein sequences revealed that MTFR1L belongs to the MTFR1 family of proteins, alongside mitochondrial-localized members MTFR1 and MTFR2, with MTFR1L being the most divergent member (*32*). MTFR1 has been implicated in regulating not only mitochondrial division, cristae architecture, and mitochondrial bioenergetics (*32*, *33*) but also cell death, as well as cancer cell metabolism and proliferation (*34*–*36*). It has recently been proposed that MTFR2 regulates mitochondrial fission during mitosis (*37*) as well as cancer cell proliferation (*38*, *39*). Furthermore, while immunoprecipitation experiments have identified MTFR1L as a potential interactor with cristae regulator protein Mic60 and the fusion protein OPA1 (*40*), its subcellular localization and functions in the regulation of mitochondrial morphology and downstream action following AMPK activation remain unknown.

To shed light on the intricate interplay between mitochondrial morphology, AMPK, and cellular homeostasis, we have investigated not only MTFR1L localization and function but also its relevance as an AMPK substrate in mammalian cells in vitro and in cortical murine neurons in vivo. We show that MTFR1L is an OMM-localized protein whose loss induces a substantial elongation and interconnection of the mitochondrial network, associated with elevated levels of the IMM fusion protein, OPA1, and accompanied by an increased number of fusion events. Last, we demonstrate that MTFR1L is a physiological substrate for AMPK in intact cells, and that phosphorylation of Ser^103^ and Ser^238^ is required to control its function in the regulation of mitochondrial morphology at steady state, and to ensure AMPK-dependent stress-induced mitochondrial fragmentation. Our findings highlight MTFR1L as a central player in the adaptation of mitochondrial dynamics to metabolic change.

## RESULTS

### MTFR1L is an OMM protein

While MTFR1L has been shown to belong to the mitochondrial-localized MTFR1 family of proteins (*32*) and to be a putative substrate for AMPK (*30*, *31*), its expression and subcellular localization have not been investigated. Analysis of protein sequence alignments revealed that MTFR1L (NM_19557.5), as well as the two putative AMPK phosphorylation sites Ser^103^ and Ser^238^ are conserved among vertebrates. Also conserved are hydrophobic residues at positions −5 and +4, and basic residues at position −3, relative to the phosphorylated serine residues (fig. S1), making both sites excellent fits to the well-established AMPK recognition motif (*41*). Predictive software (NPSA Prabi) suggested six putative α helices. In addition, according to National Center for Biotechnology Information (NCBI), human MTFR1L can be alternatively spliced to generate a shorter isoform that contains Ser^103^ but lacks Ser^238^ (NM001099627.1), which is, however, less well conserved among vertebrates and is absent in mice. Therefore, in this study, we exclusively focused on the characterization of the main longer MTFR1L isoform, which was identified in several phosphoproteomic studies as a potential AMPK substrate (*25*).

**Fig. 1.**
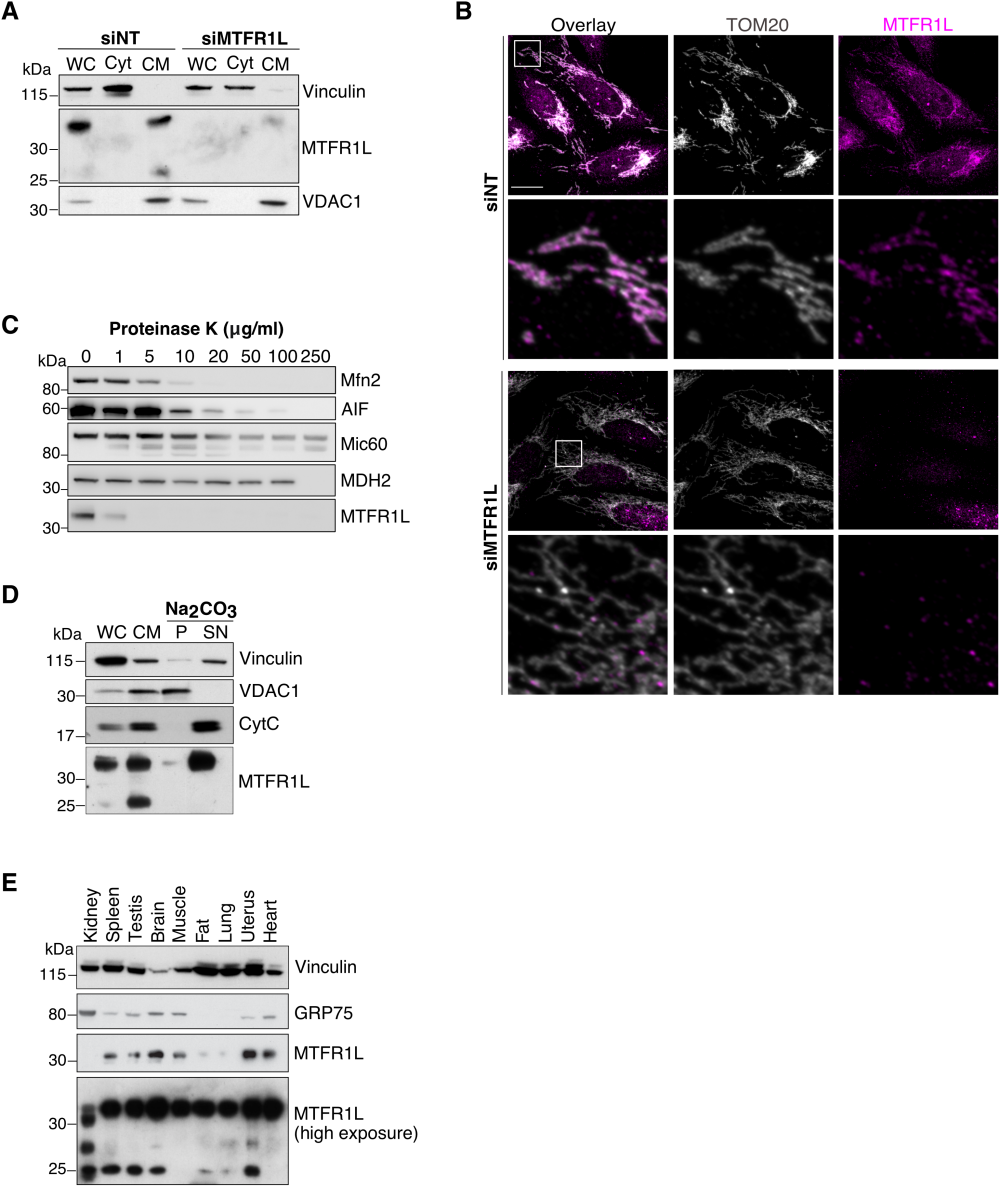
MTFR1L is a mitochondrial protein. (**A**) Immunoblot analysis of subcellular fractionation showing MTFR1L localization in HeLa cells treated with nontargeted (NT) or MTFR1L siRNA. Total whole-cell (WC) lysates were fractionated into heavy membranes containing crude mitochondria (CM) and cytosolic (Cyt) fractions. Vinculin and VDAC1 were used as cytosolic and mitochondrial markers, respectively. (**B**) Representative confocal images of MTFR1L localization from U2OS cells silenced with NT or MTFR1L siRNAs. Mitochondria and MTFR1L were labeled using anti-TOM20 and anti-MTFR1L antibodies, respectively. Scale bar, 20 μm. (**C**) Proteinase K digestion assay on crude mitochondria isolated from HeLa cells. Crude mitochondrial fractions (CMFs) were treated with increasing concentrations of proteinase K and analyzed by immunoblot. Mfn2, AIF, Mic60, and MDH2 were used as OMM, intermembrane space, IMM, and matrix markers, respectively. (**D**) Sodium carbonate treatment of CMFs isolated from HeLa cells. Whole-cell (WC), crude mitochondria (CM), pellet (P), containing membrane inserted proteins, and supernatant (SN), containing soluble proteins, fractions were analyzed by immunoblot. Vinculin was used as a whole-cell marker, Mic60 and VDAC1 were used as controls for integral membrane proteins, whereas CytC was used as control for soluble proteins. (**E**) Immunoblot analysis of MTFR1L expression in different wild-type (WT) mouse tissues. Vinculin and GRP75 were used as loading controls. Two different time exposures are represented for MTFR1L, low and high. MTFR1L was detected using the Atlas antibody (HPA027124). See also fig. S1.

We first investigated the subcellular localization of MTFR1L by both subcellular fractionation and microscopy in HeLa and U2OS cells. Immunoblot analysis of cell fractions using a commercial rabbit antibody against the long isoform of MTFR1L revealed a specific band around 38 kDa (expected mass = 32 kDa) and a second band at 25 kDa that exclusively localized to the heavy membrane fraction, which contains crude mitochondria. The specificity of the antibody binding was validated by *MTFR1L*-targeted small interfering RNA (siRNA) treatment (Fig. 1A). These results were confirmed by immunofluorescence analysis, which showed that MTFR1L exhibited a mitochondrial localization, as shown by its endogenous colocalization with the OMM marker, TOM20 (Fig. 1B). Down-regulating MTFR1L expression led to a significant reduction of the signal observed at mitochondria, showing the specificity of both the antibody and the siRNAs used (Fig. 1, A and B). We next determined the submitochondrial localization of MTFR1L by treatment of isolated mitochondria with increasing concentrations of proteinase K to sequentially degrade exposed proteins from different mitochondrial compartments (Fig. 1C). Subsequent immunoblot analysis showed that the degradation of the MTFR1L band in response to proteinase K was similar to that of the OMM resident protein Mfn2. Initial degradation of MTFR1L and Mfn2 occurred with just 1 and 5 μg/ml of proteinase K, respectively, in contrast to the intermembrane space protein, AIF, and IMM protein, Mic60, which were degraded with 10 and 20 μg/ml of proteinase K, respectively (Fig. 1C). These findings demonstrate that MTFR1L is localized to the OMM. To investigate whether MTFR1L was inserted into the OMM, we treated isolated mitochondria with sodium carbonate (pH 11) followed by centrifugation. Immunoblot analysis of the supernatant and pellet fractions revealed that MTFR1L assorted with other non-inserted proteins, such as cytochrome c, into the supernatant, indicating that MTFR1L is a labile protein associated with the cytosolic face of the OMM (Fig. 1D). However, the second band detected by the antibody at 25 kDa was not localized to either compartment, suggesting that it could represent an MTFR1L degradation product. Therefore, we decided to focus only on the long MTFR1L isoform detected at around 38 kDa through the rest of the study. Last, using mouse tissues, we showed that MTFR1L was ubiquitously expressed in all tissues examined, particularly enriched in the brain, uterus, and heart (Fig. 1E). Together, these results reveal that MTFR1L is a non-inserted mitochondrial protein that exclusively localizes to the cytosolic surface of the OMM.

### Loss of MTFR1L leads to mitochondrial elongation

Having established the OMM localization of MTFR1L, and because the other two members of the MTFR1 family (MTFR1 and MTFR2) have been proposed to regulate mitochondrial morphology via unknown mechanisms (*32*, *33*), we next investigated the contribution of MTFR1L to the regulation of mitochondrial dynamics. First, we analyzed mitochondrial morphology by confocal microscopy in U2OS and Cos-7 cells silenced for MTFR1L with two different siRNAs (fig. S2, A to K). Loss of MTFR1L led to a mitochondrial elongation phenotype in both cell lines with both siRNAs, characterized by an increase in cells harboring elongated mitochondria (fig. S2, A, C, G, and I), a decrease of mitochondrial number (fig. S2, D and J), and an increase in mitochondrial area (fig. S2, E and K). Compared to Drp1 silencing, which only led to highly elongated mitochondria (*14*), depletion of MTFR1L also induced an interconnected mitochondrial network characterized by increased mitochondrial branching and junctions in the region of interest (ROI; fig. S2F).

**Fig. 2.**
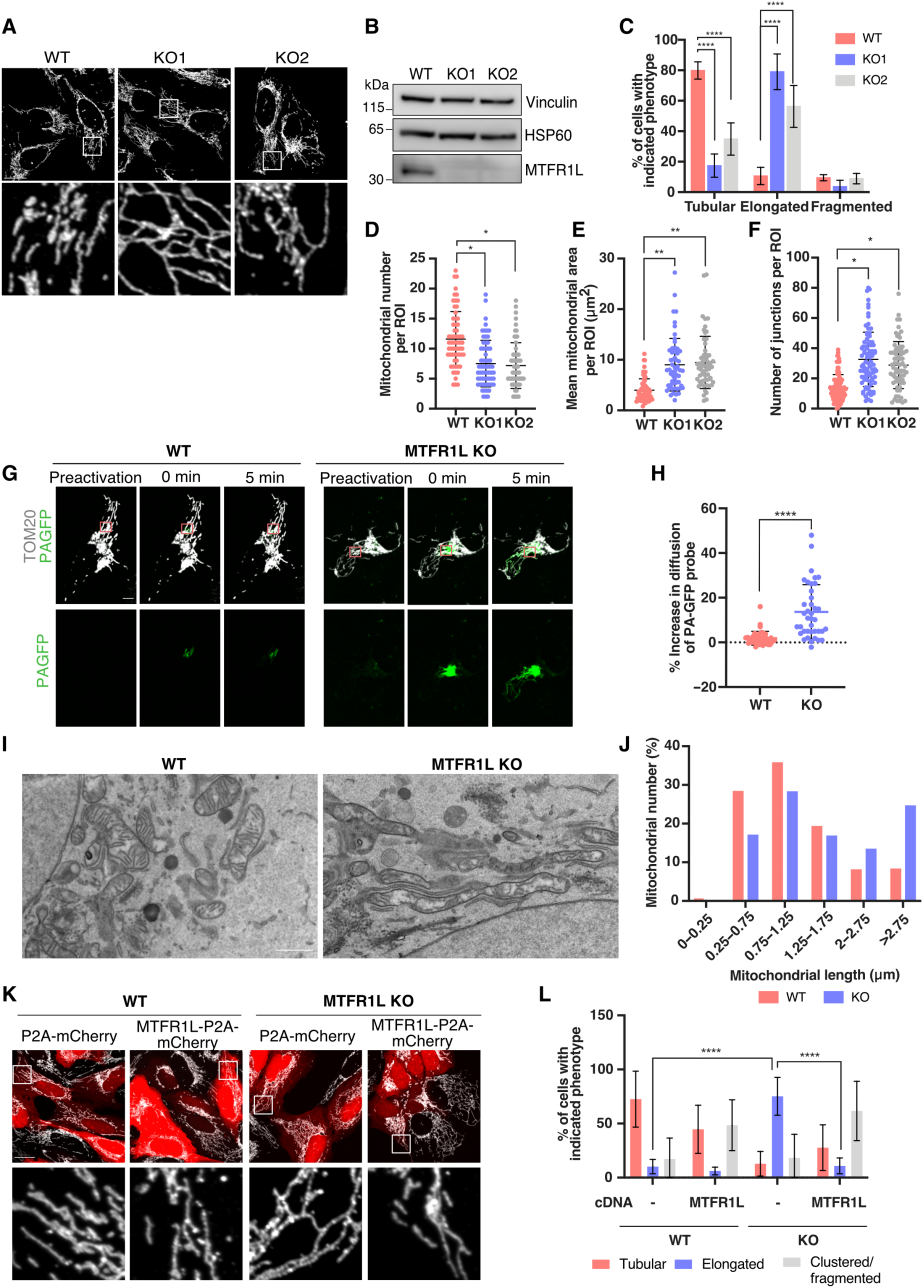
Loss of MTFR1L leads to mitochondrial elongation. (**A**) Representative confocal images of mitochondrial morphology from WT and MTFR1L KO U2OS cells. Mitochondria were labeled with an anti-TOM20 antibody. Scale bar, 20 μm. (**B**) Immunoblot analysis of MTFR1L levels in MTFR1L KO U2OS clones. Vinculin and HSP60 were used as loading controls. (**C**) Quantification of mitochondrial morphology from (A). (**D** to **F**) Mitochondrial morphology was quantified as (D) mitochondrial number, (E) mean mitochondrial area, and (F) number of junctions, per ROI. (**G**) Live-cell imaging of WT and MTFR1L KO U2OS cells overexpressing the ornithine carbamoyl transferase (OCT)–photoactivable GFP (mt-PAGFP) probe and the mitochondrial marker TOM20-mCherry. Scale bar, 10 μm. (**H**) Quantification of the OCT-PAGFP probe diffusion from (G), calculated as a ratio between OCT-PAGFP–occupied area on total mitochondria network (TOM20-mCherry) at 0 and 5 min. (**I**) Representative TEM images from WT and MTFR1L KO U2OS cells. Scale bar, 1 μm. (**J**) Quantification of TEM images showing the distribution of mitochondrial length from (I). (**K**) Representative confocal images of mitochondrial morphology of WT and MTFR1L KO U2OS cells transiently overexpressing P2A-mCherry alone or MTFR1L-P2A-mCherry. Mitochondria were labeled with an anti-TOM20 antibody. Scale bar, 20 μm. (**L**) Quantification of mitochondrial morphology from (K). All values: mean ± SD; at least three independent experiments. See also figs. S2 and S3.

To confirm these results and to examine the effects of the permanent loss of MTFR1L, CRISPR-Cas9–mediated knockout (KO) of MTFR1L was generated in U2OS cells with guide RNAs (gRNAs) targeting either exon 3 or 4. Confocal microscopy analysis revealed that both MTFR1L KO U2OS clones (KO1 and KO2) displayed around 80 and 60% of cells with elongated mitochondria, respectively (Fig. 2, A to C). In addition, similar to silenced cells, both MTFR1L KO clones exhibited an increase in mean mitochondrial area, as well as branching and junctions associated with a decrease in mitochondrial number (Fig. 2, D to F). Both MTFR1L KO clones displayed similar results, although MTFR1L KO1 cells exhibited the strongest phenotype and were used in the remainder of the study (referred to hereafter as MTFR1L KO). We further validated mitochondrial elongation in MTFR1L KO U2OS cells by overexpressing a photoactivatable green fluorescent protein (GFP) probe targeted to the mitochondrial matrix (OCT-PAGFP) (*42*) in conjunction with confocal live cell imaging analysis. As shown in Fig. 2 (G and H), MTFR1L KO cells exhibited a significant increase in the diffusion of the PA-GFP probe throughout the mitochondrial network compared to control cells, reinforcing the increased interconnectivity of the mitochondrial network previously observed. To extend further these results obtained by light microscopy, we investigated mitochondrial morphology by transmission electron microscopy (TEM). Analysis of TEM micrographs confirmed the mitochondrial elongation and interconnectivity phenotypes, as shown by an increase in organelle length and length/width aspect ratio in MTFR1L KO U2OS cells compared to control U2OS cells (Fig. 2, I and J, and fig. S2, L and M).

Last, to confirm the specificity of the mitochondrial phenotype induced by loss of MTFR1L, we performed rescue experiments using an MTFR1L-P2A-mCherry plasmid, which enabled the visualization of MTFR1L-expressing cells. Reexpression of MTFR1L in MTFR1L KO U2OS cells rescued mitochondrial elongation, with an increase in mitochondrial number and a decrease in mitochondrial area, to levels similar to control cells (Fig. 2, K and L, and fig. S2, N to P). Overexpression of MTFR1L in wild-type (WT) cells also led to mitochondrial fragmentation and perinuclear clustering in 44% of the cells (Fig. 2L). We also generated an MTFR1L mutant lacking the first helix comprising the first 28 amino acids of the N-terminal domain. In contrast to WT MTFR1L, MTFR1L^Δ1–28^ was mainly expressed in the cytosol and, therefore, unable to rescue mitochondrial elongation observed in MTFR1L KO U2OS cells (fig. S3). These results indicate that the N-terminal domain of MTFR1L is important not only for its localization but also for its capacity to regulate mitochondrial morphology. Together, these results demonstrate that loss of MTFR1L leads to a specific mitochondrial elongation phenotype, which indicates its contribution to the regulation of mitochondrial morphology.

### Depletion of MTFR1L increases OPA1 levels and mitochondrial fusion events

**Fig. 3.**
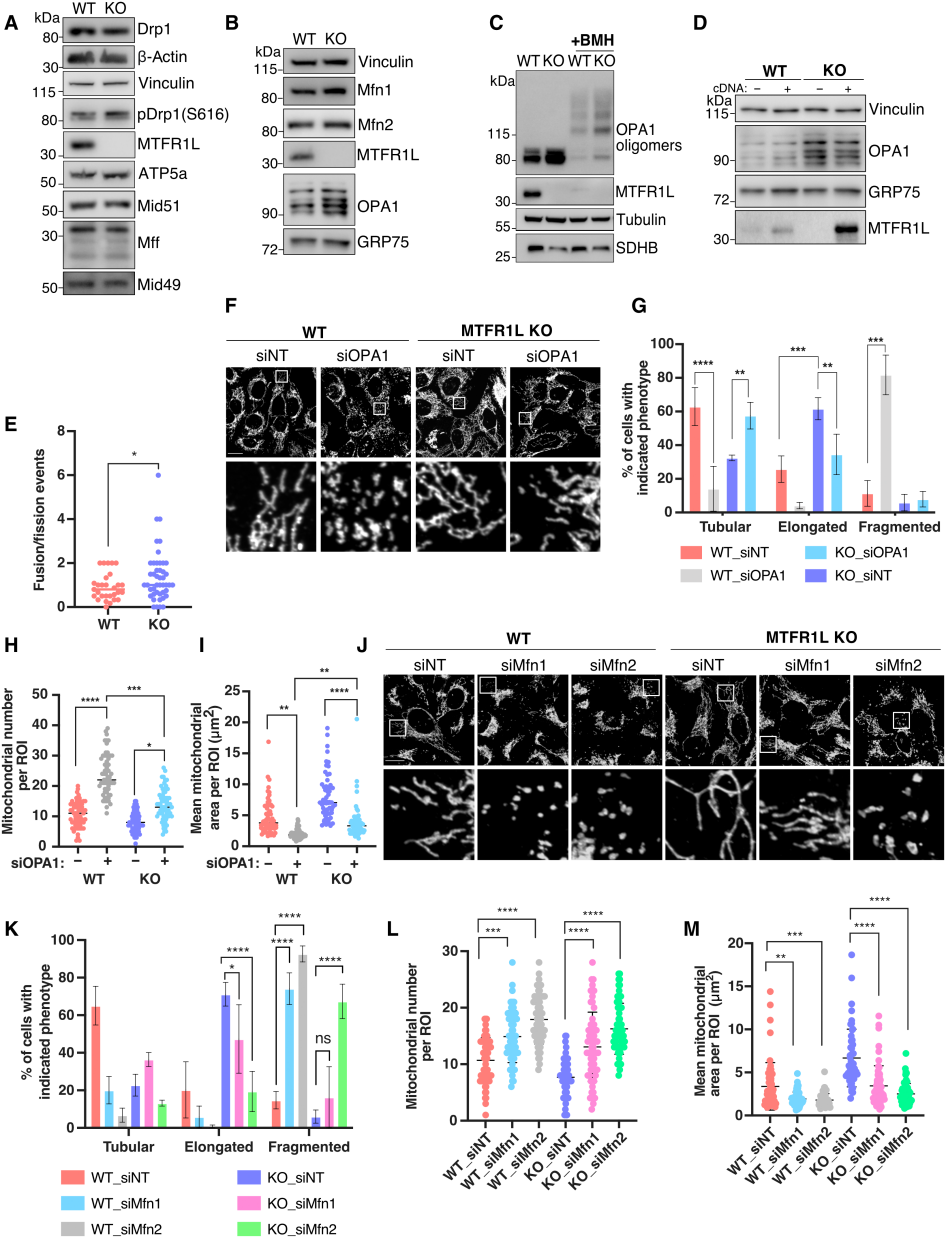
Mitochondrial elongation induced by MTFR1L loss is due to increased mitochondrial fusion. (**A** and **B**) Immunoblots of proteins related to mitochondrial (A) division and (B) fusion, from WT and MTFR1L KO U2OS cells. Vinculin, β-actin, ATP5a, and GRP75 were used as loading controls. (**C**) Immunoblots of OPA1 oligomers from WT and MTFR1L KO U2OS cells treated with dimethyl sulfoxide (DMSO) or cross-linker 1,6-bismaleimidohexane (BMH). Tubulin and SDHB were used as loading controls. (**D**) Immunoblots of OPA1 levels from WT and MTFR1L KO U2OS cells transiently overexpressing empty vector pcDNA 3+ (−) and pcDNA 3-MTFR1L (+). Vinculin and GRP75 were used as loading controls. (**E**) Quantification of mitochondrial fusion and division events from WT and MTFR1L KO U2OS cells transiently overexpressing the mitochondrial marker GFP-OMP25. Representative quantification of the ratio between fusion and fission events per 625-μm^2^ ROI over a 5-min period is shown as a scatterplot. (**F**) Representative confocal images of WT and MTFR1L KO U2OS cells silenced with indicated siRNAs. Mitochondria were labeled using an anti-TOM20 antibody. (**G**) Quantification of mitochondrial morphology from (F). (**H** and **I**) Mitochondrial morphology was quantified as (H) mitochondrial number and (I) mean mitochondrial area, per ROI. (**J**) Representative confocal images of WT and MTFR1L KO U2OS cells silenced with indicated siRNAs. Mitochondria were labeled using an anti-TOM20 antibody. (**K**) Quantification of mitochondrial morphology from (J). (**L** and **M**) Mitochondrial morphology was quantified as (L) mitochondrial number and (M) mean mitochondrial area, per ROI. All scale bars, 20 μm. All values: mean ± SD; at least three independent experiments. See also figs. S4 to S8.

Mitochondrial elongation can arise from either a defect in mitochondrial division or an increase in organelle fusion (*1*). To gain a deeper understanding of the underlying mechanism and to determine which process led to mitochondrial elongation in MTFR1L KO cells, we first investigated potential defects in mitochondrial division, by assessing the levels of various effectors of mitochondrial fission by immunoblot analyses. We observed no changes in fission-associated proteins in MTFR1L KO U2OS cells, or in MTFR1L U2OS– and Cos-7–silenced cells, including the main driver of mitochondrial division, Drp1, or its receptors MFF and MiD49/51 (Fig. 3A and fig. S4, A to E). We next examined the capacity of Drp1 to be recruited to mitochondrial membranes, a key step in mitochondrial division. Subcellular fractionation and confocal imaging of Drp1 subcellular distribution in MTFR1L KO U2OS cells revealed no defect in Drp1 recruitment to mitochondria (fig. S5, A and B). Last, to investigate the capacity of mitochondria to divide in cells lacking MTFR1L, we promoted Drp1-dependent mitochondrial division either by treatment with the mitochondrial uncoupler carbonyl cyanide 3-chlorophenylhydrazone (CCCP; fig. S5, C and D) or by overexpressing the pro-fission factor mitochondrial anchored protein ligase (MAPL; fig. S5, E and F) (*43*). Both conditions rescued the mitochondrial elongation phenotype and led to similar mitochondrial fragmentation as seen in control cells. We conclude that cells lacking MTFR1L retain their ability and machinery to trigger mitochondrial division. Thus, these results indicate that while MTFR1L loss leads to mitochondrial elongation, this phenotype is not due to either a defect in Drp1 recruitment to mitochondria or a lack of mitochondrial division.

We next investigated whether the loss of MTFR1L was instead associated with an increase in mitochondrial fusion. Immunoblot analysis revealed that loss of MTFR1L led to increased levels of all different OPA1 isoforms, the main actor of IMM fusion, but not of the outer membrane fusion regulators Mfn1 and Mfn2 (Fig. 3B and fig. S6, A to E). In addition, cross-linking experiments also showed an increase in OPA1 oligomer levels (Fig. 3C), suggesting that increased fusion because of OPA1 levels underlies the mitochondrial elongation phenotype observed in cells lacking MTFR1L. There was no increase in *Opa1* mRNA content in MTFR1L KO U2OS cells, as opposed to MTFR1L-silenced U2OS cells (fig. S6, F and G), suggesting a posttranscriptional mechanism accounting for elevated OPA1 levels, at least in MTFR1L KO cells. We demonstrated the specificity of this phenotype by reexpressing MTFR1L, which led to a significant rescue of the increased OPA1 protein levels observed (Fig. 3D and fig. S6H). However, transient expression of non–mitochondrial-localized MTFR1L^Δ1–28^ mutant in MTFR1L KO U2OS cells did not rescue OPA1 levels (fig. S3A), indicating the critical role of the mitochondrial localization of MTFR1L to control OPA1 levels.

We next performed live cell confocal microscopy analysis to quantify fusion and fission events in U2OS cells expressing the GFP-OMP25 marker to label mitochondria. In contrast to mitochondrial division events, quantitative analysis revealed an increased number of fusion events and of the ratio of fusion to fission events in MTFR1L KO cells (Fig. 3E and fig. S7, A and B), confirming the excessive fusion and suggesting a role for MTFR1L in suppressing mitochondrial fusion. To extend these findings, we silenced the main proteins involved in mitochondrial fusion (OPA1, Mfn1, and Mfn2) and examined how this affected mitochondrial elongation induced by MTFR1L loss. Loss of either OPA1, Mfn1, or Mfn2 rescued the mitochondrial elongation observed in MTFR1L KO U2OS cells. This was associated with a reduction of cells harboring a hyperfused phenotype, an increase in mitochondrial number, and a reduction in mitochondrial area (Fig. 3, F to M, and fig. S7, C and D). Last, we hypothesized that if MTFR1L was negatively regulating fusion, as already proposed for Fis1 (*44*), its silencing in Drp1 KO cells, unable to divide their mitochondria, should lead to increased fusion events and induce interconnected mitochondria. As shown in fig. S7E, loss of MTFR1L in Drp1 KO cells further shifted the specific elongated mitochondria phenotype toward a branched and interconnected mitochondrial phenotype, similar to that found in MTFR1L KO cells (fig. S7, E to G). Thus, these results indicate that the loss of MTFR1L in Drp1 KO cells induced further mitochondrial fusion.

Mitochondrial morphology and increased levels of OPA1 are critical to enhance cell survival by acting on different mitochondrial features including mitochondrial cristae architecture (*45*, *46*) and resistance to apoptosis (*47*). TEM analysis revealed that MTFR1L KO U2OS cells exhibited an increase in mitochondrial cristae tightness characterized by a decrease in cristae width, similar to the phenotype observed upon overexpression of OPA1 (fig. S8, A and B) (*48*). In addition, immunoblot and immunofluorescence analyses showed that MTFR1L KO U2OS were also resistant to cell death induced by ABT 737 and actinomycin D treatment characterized by decreased levels of cleaved caspase 3 (fig. S8C) and a decrease of cells with cytosolic cytochrome c (fig. S8, D and E) compared to control-treated U2OS cells, two hallmarks of apoptosis induction. Last, silencing OPA1 induced the presence of cytochrome c in the cytosol in MTFR1L KO–treated cells (fig. S8, D and E), corroborating the OPA1-dependent mitochondrial hyperfusion observed in cells lacking MTFR1L. Overall, these findings show that loss of MTFR1L increases levels of OPA1 and mitochondrial fusion and suggest that MTFR1L behaves as an anti-fusion protein, negatively regulating mitochondrial fusion events by controlling OPA1 levels.

### AMPK-dependent MTFR1L phosphorylation controls mitochondrial morphology

**Fig. 4.**
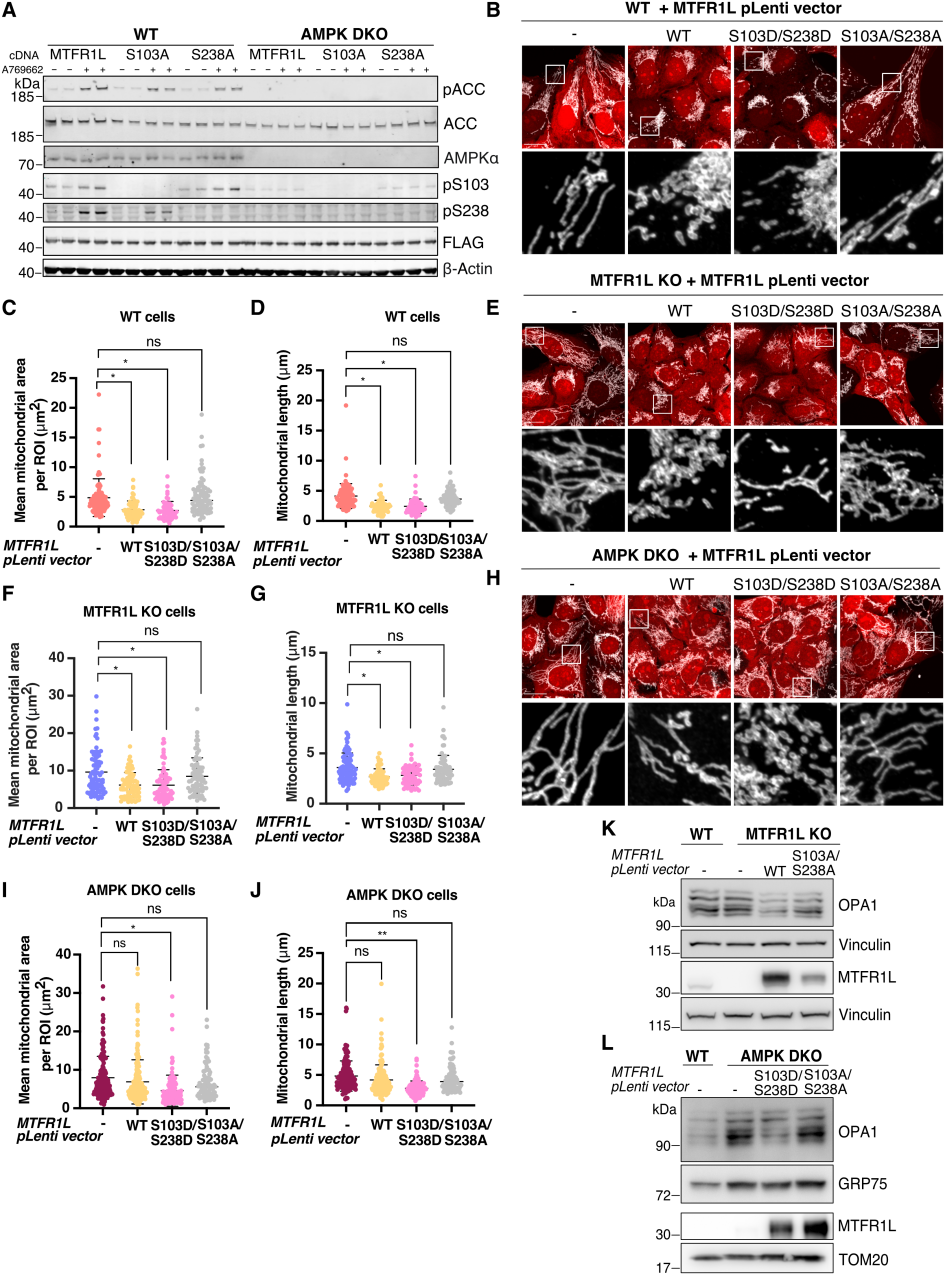
AMPK-dependent phosphorylation of MTFR1L controls mitochondrial morphology. (**A**) Immunoblot analysis of indicated proteins and of MTFR1L phosphorylation in WT and AMPK-α1^−/−^-α2^−/−^ DKO U2OS cells, transiently overexpressing FLAG-WT MTFR1L^WT^, FLAG-MTFR1L^S103A^, or FLAG-MTFR1L^S238A^ phosphomutants, and treated with DMSO (−) or AMPK activator, A769662 (+), for 1 hour at 200 μM. Specific rabbit anti-MTFR1L phosphospecific antibodies were used to detect the two distinct phosphorylations at Ser^103^ and Ser^238^. Anti-FLAG was used to confirm overexpression of the different constructs, and β-actin was used as a loading control. Samples from two different experiments were loaded side by side. (**B**, **E**, and **H**) Representative confocal images of (B) WT, (E) MTFR1L KO, and (H) AMPK-α1α2 DKO U2OS cells stably expressing P2A-mCherry alone (−), MTFR1L^WT^-P2A-mCherry, double phosphomimetic MTFR1L^S103D/S238D^-P2A-mCherry, or double phosphomutant MTFR1L^S103A/S238A^-P2A-mCherry. Mitochondria were labeled with an anti-TOM20 antibody. (**C**, **D**, **F**, **G**, **I**, and **J**) Mitochondrial morphology was quantified as (C, F, and I) mean mitochondrial area and (D, G, and J) mitochondrial length, per ROI from (B), (E), and (H). (**K**) Immunoblots of OPA1 levels from WT and MTFR1L KO U2OS cells stably expressing P2A-mCherry vector alone (−), MTFR1L^WT^-P2A-mCherry, or MTFR1L^S103A/S238A^-P2A-mCherry. Vinculin was used as loading control. (**L**) Immunoblots of OPA1 levels from WT and AMPK-α1α2 DKO U2OS cells transiently expressing P2A-mCherry vector alone (−), MTFR1L^S103D/S238D^-P2A-mCherry, or MTFR1L^S103A/S238A^-P2A-mCherry. GRP75 and TOM20 were used as loading controls. All scale bars, 20 μm. All values: mean ± SD; at least three independent experiments. See also fig. S9.

Recent phosphoproteomic studies identified MTFR1L as a potential substrate of the master energy regulator AMPK and revealed two putative phosphorylated residues: Ser^103^ and Ser^238^ (*30*, *31*). To experimentally validate MTFR1L and the two phosphorylation sites as physiological AMPK targets, we generated two phospho-specific antibodies against MTFR1L pSer^103^ and pSer^238^. We also generated MTFR1L nonphosphorylatable mutant constructs by mutating these serine residues to alanine (S103A and S238A), and transiently expressed them in parallel with WT MTFR1L complementary DNA (cDNA), in both WT and AMPK-α1α2 double KO (DKO) U2OS cells generated using the Cas9 (D10A) double nickase system (*49*). Next, we analyzed MTFR1L phosphorylation under steady-state conditions and following AMPK activation with A-769662, a well-characterized AMPK activator (*50*, *51*). As expected in WT cells, A-769662 treatment led to AMPK activation, confirmed by the phosphorylation of acetyl coenzyme A carboxylase (ACC; Fig. 4A). Immunoblot analysis revealed MTFR1L phospho-specific bands, detected with antibodies raised against both MTFR1L^pS103^ and MTFR1L^pS238^, in cells expressing MTFR1L^WT^ under basal conditions, which markedly increased in intensity upon A-769662 treatment (Fig. 4A). Furthermore, phosphorylation of MTFR1L at Ser^103^ or Ser^238^ was abolished in cells expressing MTFR1L^S103A^ and MTFR1L^S238A^, respectively, confirming the specificity of the antibodies and the phosphorylation of MTFR1L on these respective residues. Last, in contrast to WT cells, bands corresponding to phosphorylation of either MTFR1L Ser^103^ or Ser^238^ were not detected in AMPK-α1/α2 DKO U2OS cells, whether or not treated with A-769662, showing that MTFR1L phosphorylation at these sites was AMPK dependent. Together, these data revealed that MTFR1L is an AMPK target phosphorylated at both Ser^103^ and Ser^238^.

Because our results indicated a contribution of MTFR1L in the regulation of mitochondrial fusion, and recent studies have highlighted the roles for AMPK-dependent phosphorylation of MFF (*29*) and ARMC10 (*30*) in mitochondrial dynamics, we hypothesized that MTFR1L phosphorylation would trigger mitochondrial morphological changes in an AMPK-dependent manner. To investigate this hypothesis, we stably expressed MTFR1L^WT^, the potentially active double phosphomimetic mutant MTFR1L^S103D/S238D^ or the double nonphosphorylatable mutant MTFR1L^S103A/S238A^ in WT, MTFR1L KO, and AMPK-α1α2 DKO U2OS cells, and analyzed mitochondrial morphology (Fig. 4, B to J, and fig. S9, A to F). Confocal microscopy showed that expression of both MTFR1L^WT^ and phosphomimetic MTFR1L^S103D/S238D^ in WT U2OS cells led to fragmentation of the mitochondrial network, characterized by decreased mean mitochondrial area and length, along with mitochondrial perinuclear clustering (Fig. 4, B to D, and fig. S9, A and B). In contrast, expression of the double nonphosphorylatable mutant MTFR1L^S103A/238A^ had no effect on mitochondrial morphology (Fig. 4, B to D, and fig. S9B).

In MTFR1L KO U2OS cells, expression of both WT and phosphomimetic MTFR1L^S103D/238D^ rescued the mitochondrial hyperfusion phenotype and induced a fragmented and perinuclear clustered mitochondrial phenotype (Fig. 4, E to G, and fig. S9, C and D). However, expression of double nonphosphorylatable mutant MTFR1L^S103A/S238A^ had no impact on mitochondrial morphology (Fig. 4, E to G, and fig. S9, C and D), indicating that MTFR1L phosphorylation is required to regulate mitochondrial dynamics. To elucidate the role of AMPK in this process, similar experiments were performed in AMPK-α1/α2 DKO U2OS cells, which exhibited elongated mitochondria compared to control U2OS cells (*29*) (compare elongated mitochondria in Fig. 4, H versus B). Compared to WT and MTFR1L KO U2OS cells, expression of MTFR1L^WT^ in AMPK-α1/α2 DKO U2OS cells did not induce marked mitochondrial fragmentation, nor a change in mean mitochondrial length and area (Fig. 4, H to J, and fig. S9, E and F). However, expression of phosphomimetic MTFR1L^S103D/S238D^ still led to mitochondrial morphology remodeling and a shift in the mitochondrial network toward mitochondrial fragmentation and perinuclear clustering compared to all other conditions (Fig. 4, H to J, and fig. S9, E and F).

To further elucidate the role of MTFR1L phosphorylation in regulating mitochondrial morphology and fusion, we analyzed all OPA1 isoform levels in MTFR1L KO and AMPK-α1/α2 DKO U2OS cells expressing our different MTFR1L constructs. In contrast to the expression of double nonphosphorylatable mutant MTFR1L^S103A/S238A^, stable expression of MTFR1L^WT^ decreased OPA1 levels in MTFR1L KO U2OS cells (Fig. 4K and fig. S9G). In addition, we observed an increase in OPA1 protein levels in AMPK-α1/α2 DKO U2OS, which was rescued by stably expressing MTFR1L^S103D/238D^ but not the MTFR1L^S103A/S238A^ mutant, similar to mitochondrial morphology analysis (Fig. 4L and fig. S9H). Thus, these results highlight that phosphorylation of MTFR1L is sufficient to induce mitochondrial fragmentation and indicate that the AMPK-MTFR1L axis is critical to drive mitochondrial network remodeling via the control of OPA1 levels.

### MTFR1L is required for AMPK-dependent mitochondrial fragmentation

**Fig. 5.**
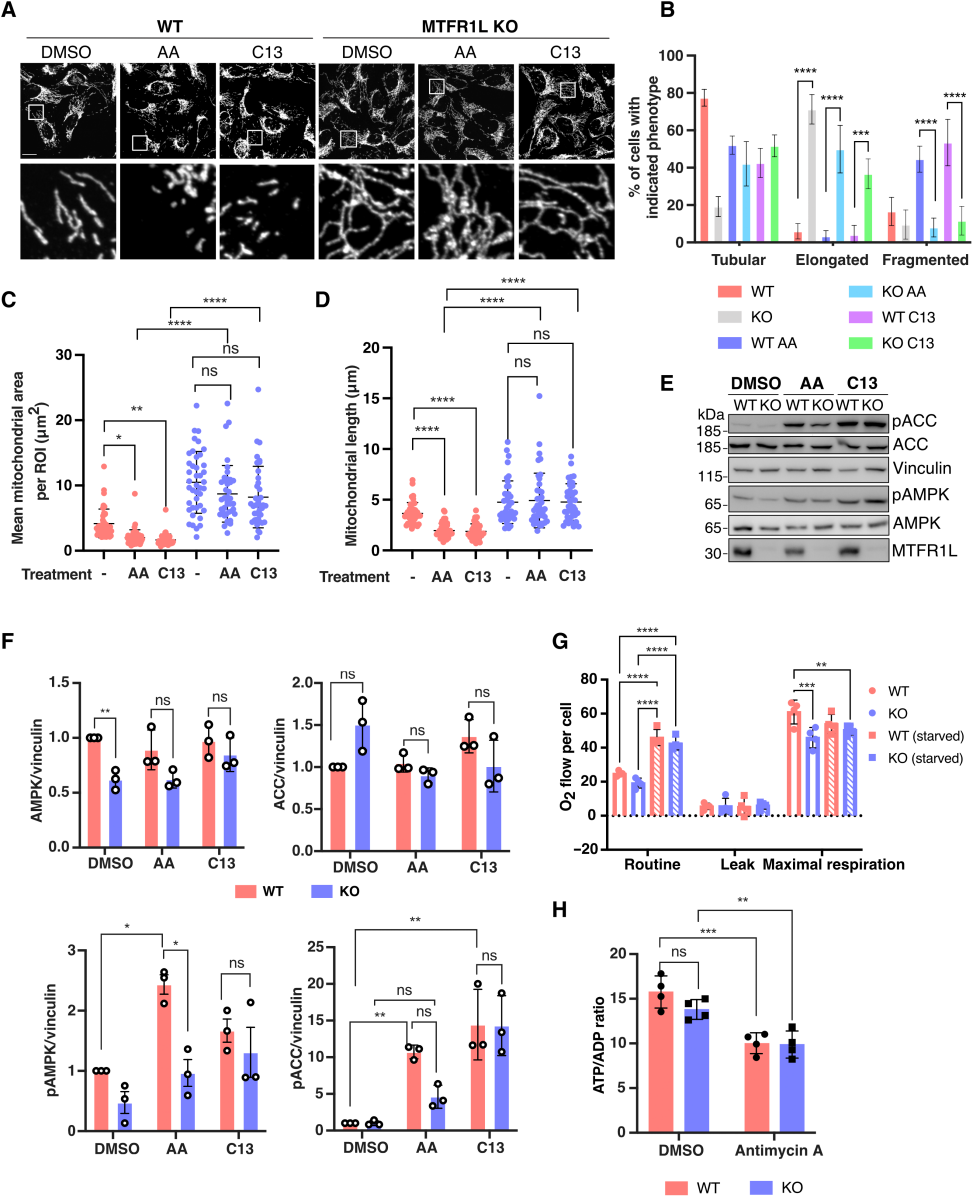
MTFR1L is required for AMPK-driven mitochondrial fragmentation. (**A**) Representative confocal images of WT and MTFR1L KO U2OS cells treated with DMSO, 10 μM antimycin A (AA), or the allosteric AMPK activator C13 for 1 hour. Mitochondria were labeled with an anti-TOM20 antibody. Scale bar, 20 μm. (**B**) Quantification of mitochondrial morphology from (A). (**C** and **D**) Mitochondrial morphology was quantified as (C) mean mitochondrial area and (D) mitochondrial length, per ROI. (**E**) Immunoblot analysis of indicated proteins corresponding to (A) showing AMPK activation in WT and MTFR1L KO U2OS cells, treated with DMSO, 10 μM antimycin A (AA), or the allosteric AMPK activator C13 for 1 hour. Vinculin was used as a loading control. (**F**) Quantification of levels of the indicated proteins from (E). Signal intensities were quantified by densitometry and normalized to loading controls. *N* = 3 independent experiments. (**G**) High-resolution respirometry analyses performed in intact WT and MTFR1L KO U2OS cells using Oroboros instrument. ROUTINE: cellular basal oxygen consumption rate (pmol O_2_/s) per million cells in supplemented medium or lacking glucose (starved for 4 hours). Leak is the nonphosphorylating respiration in the presence of ATP synthase inhibitor oligomycin. ETS: maximal respiration rate in the presence of the uncoupler CCCP. *N* = 3 independent experiments. (**H**) Quantification of the ATP/ADP ratio determined by bioluminescence assay from WT and MTFR1L KO U2OS cells treated with DMSO or antimycin A. *N* = 3 independent experiments. All values: mean ± SD or SEM; at least three independent experiments. See also figs. S10 and S11.

AMPK has evolved not only as an energy sensor and master regulator of cellular metabolism but also as a critical regulator of mitochondrial function including biogenesis, bioenergetics, and dynamics (*28*). During ETC-induced mitochondrial dysfunction, the decreased ATP/adenosine diphosphate (ADP) ratio activates AMPK, which phosphorylates MFF, thereby increasing Drp1 recruitment and leading to mitochondrial division, which may facilitate clearance of dysfunctional mitochondria (*29*). To investigate whether MTFR1L was also required for AMPK-dependent morphological changes during stress, MTFR1L KO and silenced U2OS cells were treated with inhibitors of respiratory complex I (rotenone) and complex III (antimycin A), and mitochondrial morphology was monitored (Fig. 5, A to D). Antimycin A treatment of WT U2OS cells activated AMPK, as monitored by its phosphorylation and that of its substrate ACC (Fig. 5, E and F), which was accompanied by mitochondrial fragmentation compared to control untreated cells (Fig. 5, A to D). However, antimycin A treatment of MTFR1L KO U2OS cells failed to rescue mitochondrial hyperfusion or to induce mitochondrial fragmentation (Fig. 5, A to D). These results were also confirmed in MTFR1L-silenced U2OS where neither ETC inhibitors (antimycin A and rotenone) induced mitochondrial fragmentation compared to siNT-treated cells (figs. S10, A and B, and S11, A and B). Unexpectedly, immunoblot analysis revealed that treatment with these ETC inhibitors of both MTFR1L KO and silenced U2OS cells decreased AMPK activation, as indicated by decreased levels of phospho-ACC and phospho-AMPK compared to similarly treated control cells (Fig. 5, E and F, and figs. S10, C and D, and S11, C and D). These results suggest that mitochondrial hyperfusion observed in cells lacking MTFR1L could protect from ETC-induced dysfunction by maintaining a sufficient level of ATP production to maintain AMPK inactivated. To test this hypothesis, we analyzed mitochondrial bioenergetics in MTFR1L KO U2OS cells at steady states or during glucose starvation. Unexpectedly, neither oxygen consumption nor ATP/ADP ratio measured via luciferin bioluminescence was impaired in MTFR1L KO U2OS cells compared to control (Fig. 5, G and H). These results suggest that the cause of AMPK activation dampening observed in MTFR1L KO cells is not due to fluctuations in the ATP/ADP ratio level.

Last, to confirm that the lack of mitochondrial network remodeling in cells lacking MTFR1L was not due to decreased AMPK activation, MTFR1L KO U2OS cells were treated with compound 13 (C13), which is a cell-permeable prodrug that is converted inside cells into the AMP mimetic C2, a potent allosteric activator of AMPK (*52*, *53*). While C13 led to a similar level of AMPK activation in both WT and MTFR1L KO cells (Fig. 5, E and F), it failed to reduce mitochondrial hyperfusion or to induce mitochondrial fragmentation in MTFR1L KO U2OS cells as compared to WT U2OS-treated cells (Fig. 5, A to D).

Together, these results demonstrate that MTFR1L is critical to remodel the mitochondrial network upon stress-induced AMPK activation, to sustain cellular homeostasis, and to drive cell fate decisions.

### MTFR1L phosphorylation regulates mitochondrial morphology in vivo

Given the prevalence of MTFR1L expression in the brain (Fig. 1E), which is supported by publicly available RNA sequencing and in situ hybridization datasets (fig. S12), we evaluated its function in neurons by investigating the role of MTFR1L and its phosphorylation in the regulation of mitochondrial morphology in the axons of layer 2/3 cortical pyramidal neurons (PNs) of mice in vitro and in vivo. Axonal mitochondria are ideal to test the function of pro-fission or anti-fusion protein candidates because (i) these mitochondria are very small (~1 μm length) and uniform in size (*54*) and (ii) we have shown recently that this size is maintained through high levels of MFF-Drp1–mediated fission (*54*).

**Fig. 6.**
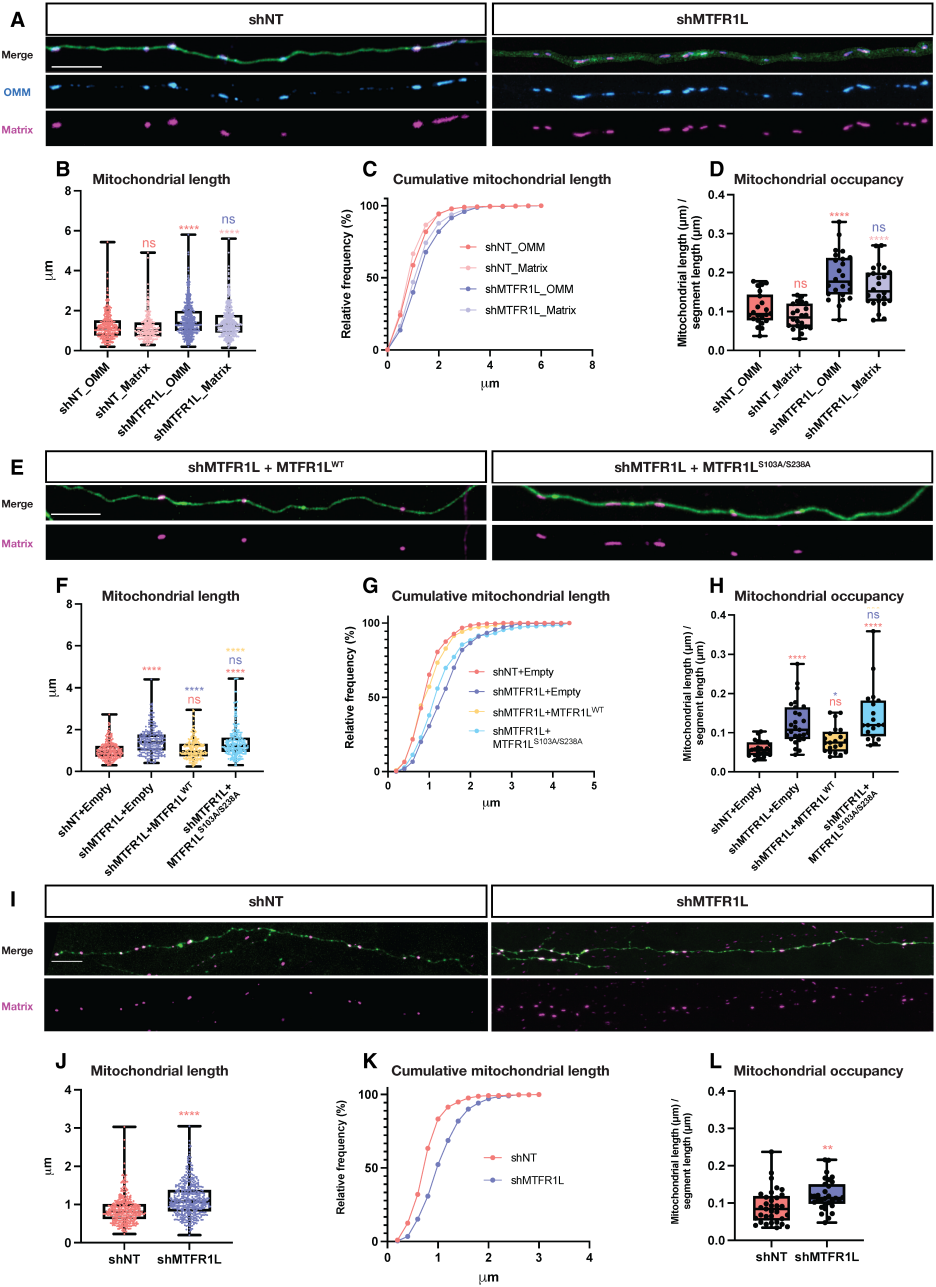
Knockdown of MTFR1L in mouse cortical PNs leads to elongated mitochondria and increased mitochondrial density in the axon. (**A**) Representative confocal images of axons from small hairpin (sh) nontargeted (shNT) or MTFR1L (shMTFR1L) expressing layer 2/3 PNs at 14 days in vitro (DIV). Axonal mitochondrial morphology was visualized by cortical ex utero electroporation of a combination of (i) genetically encoded mitochondrial markers: OMM-targeted mCherry (ActA-mCherry), matrix-targeted mtagBFP2 (mito-mTagBFP2), or matrix-targeted YFP (mito-YFP), (ii) cytoplasmic filler tdTomato or mtagBFP2, and (iii) either control shNT or shMTFR1L. Individual E15.5 brains electroporated were dissociated and cocultured. (**B** to **D**) Mitochondrial morphology was quantified from (A) as (B) mean mitochondrial length, (C) cumulative mitochondrial length, and (D) mitochondrial occupancy in the axons measured by the cumulative mitochondrial length in an axonal segment divided by the total segment length. (**E**) Representative confocal images of shMTFR1L-expressing DIV14 axons electroporated with mito-mTagBFP2 and tdTomato, and overexpressing either WT-MTFR1L^WT^ or double phosphomutant MTFR1L^S103D/S238D^. (**F** to **H**) Mitochondrial morphology was quantified from (E) as (F) mean mitochondrial length, (G) cumulative mitochondrial length, and (H) mitochondrial occupancy in the axons. (**I**) Representative confocal images of contralateral axonal projections from shNT- or shMTFR1L-treated layer 2/3 PNs at postnatal day 21 (P21). Axonal mitochondrial morphology was visualized by cortical in utero electroporation of mito-YFP and tdTomato together with shNT or mitochondrial matrix (matrix-DsRed) probe and cytoplasmic filler Venus together with shMTFR1L. (**J** to **L**) Mitochondrial morphology was quantified from (I) as (J) mean mitochondrial length, (K) cumulative mitochondrial length, and (L) mitochondrial occupancy in the axons. All scale bars, 10 μm. All values: mean ± SD; at least three independent experiments. See also figs. S12 and S13.

Initial knockdowns were performed in vitro using ex utero electroporation to sparsely incorporate short hairpin RNA (shRNA; shMTFR1L or control shRNA) and overexpress genetically encoded OMM, mitochondrial matrix, and cytoplasmic targeting fluorophores into layer 2/3 cortical PNs (Fig. 6). Control experimental conditions were achieved by coculturing neurons electroporated with control shRNA with neurons electroporated with MTFR1L shRNA (co-electroporation with different combinations of cell filler and mitochondrial markers; fig. S13) to minimize confounding variables linked to culture conditions or other batch effects. Similar to our results obtained in U2OS and Cos-7 cells, confocal microscopy analysis revealed that knockdown of MTFR1L led to mitochondrial elongation in the axons of layer 2/3 cortical PNs in vitro (Fig. 6, A to D). In shMTFR1L-treated axons, the mitochondrial network, labeled with OMM and mitochondrial matrix markers, was characterized by an increase in mitochondria length and occupancy in axons, compared to shNT control (Fig. 6, B to D), indicating that loss of MTFR1L led to elongation of both mitochondrial compartments. Rescue experiments were then performed and expressing MTFR1L^WT^ together with shMTFR1L rescued mitochondrial morphology to lengths similar to controls (Fig. 6, E to H). However, co-electroporation of the MTFR1L shRNA with double phosphomutant MTFR1L^S103A/S238A^ construct was unable to rescue mitochondrial elongation as measured by mitochondrial length or axonal occupancy (Fig. 6, E to H), indicating that these AMPK-dependent phosphorylation sites are critical for the function of MTFR1L in the maintenance of mitochondrial size in axons of layer 2/3 PNs.

To assess MTFR1L knockdown in vivo, we electroporated shNT or shMTFR1L along with a cell fill marker and matrix-targeted fluorophore in E15.5 pups that were then perfused at P21, and axonal mitochondrial morphology was analyzed by confocal imaging of contralateral axonal projections. Knockdown of MTFR1L led to mitochondrial elongation in axonal segments projecting to contralateral layer 2/3 (Fig. 6, I to K), to a very similar degree than in vitro, and was associated with an increase of mitochondria occupancy in the axon (Fig. 6L). Together, these findings support a role for MTFR1L via its phosphorylation in maintaining axonal mitochondrial morphology in vitro and in vivo in cortical PNs.

## DISCUSSION

Mitochondrial dynamics entail a series of complex events that are fulfilled by major GTPase proteins. Over the years, numerous groups have unraveled and investigated previously unidentified proteins potentially involved in regulating these processes (*4*). In this study, we present the first characterization of MTFR1L, a newly recognized OMM protein regulating mitochondrial morphology. We demonstrate that MTFR1L is a genuine AMPK substrate and show that two residues phosphorylated by AMPK are critical for the function of MTFR1L in controlling mitochondrial dynamics, in both mammalian cells in vitro and murine neurons in vivo. Together, these results reveal MTFR1L as a central player in adapting mitochondrial dynamics to metabolic alterations.

Through gene editing techniques, we show that acute or chronic loss of MTFR1L through siRNA or CRISPR-Cas9–mediated technologies, respectively, leads to a robust mitochondrial hyperfusion phenotype that is conserved among different mammalian cell lines as well as in axons of layer 2/3 cortical PNs of mice. Mechanistic studies reveal that the loss of MTFR1L does not affect Drp1 mitochondrial recruitment, nor the capacity of mitochondria to fragment upon enforced Drp1-dependent fragmentation, excluding a role of this protein in the division machinery per se. On the other hand, live-cell imaging and immunoblot analyses reveal a shift toward increased fusion events and increased levels of the IMM fusion regulator OPA1, respectively. In contrast to MFF- or Drp1-silenced cells (*55*), OPA1 silencing rescued the mitochondrial phenotypes in MTFR1L KO cells, including mitochondrial elongation and resistance to cell death. Last, rescue experiments revealed that the N-terminal domain of MTFR1L is required not only for MTFR1L mitochondrial localization but also for its capacity to regulate mitochondrial morphology. Collectively, these results indicate a role for MTFR1L in the negative regulation of mitochondrial fusion, rather than promoting division. Therefore, this study opens up avenues for understanding how the MTFR1 family regulates mitochondrial dynamics. Future investigations will be required to elucidate whether (i) the other MTFR1 members (MTFR1/MTFR2) regulate mitochondrial morphology through similar mechanisms or by directly controlling the mitochondrial fission process and (ii) MTFR1, MTFR2, and MTFR1L have diverged during evolution and acquired specialized roles to control mitochondrial dynamics in response to different stimuli.

Dynamic mitochondrial shape transitions are required not only to ensure proper mitochondrial function but also to respond to cellular needs, to adapt to different cellular stress and metabolic state, and to subsequently control cell fate decisions (*2*). For example, during mild stress, nutrient starvation, or mTORC1 inhibition, functional mitochondria elongate their network to sustain mitochondrial functions, increase bioenergetics, and protect themselves from autophagic degradation, ultimately enhancing cell survival (*56*–*58*). In contrast, upon severe mitochondrial stress, mitochondrial fragmentation is increased to allow autophagic degradation of damaged mitochondria (*59*), or ultimately to enhance apoptosis (*60*). Here, we propose that loss of MTFR1L leads to enhanced mitochondrial fusion via the increased protein levels of all different OPA1 isoforms. Up until now, few studies have documented a relative posttranscriptional up-regulation of OPA1 levels associated with a cellular adaptation to maintain homeostasis and survival. For example, during metabolic stress induced by cell starvation, OPA1 oligomerizes, leading to tightness of the cristae and mitochondrial elongation, to increase mitochondrial respiration to prevent cell death (*61*). In addition, OPA1 levels (and in particular increased OPA1 oligomers) have recently been proposed to be critical to maintain mitochondrial ultrastructure and cellular homeostasis upon antimycin A treatment (*48*). Loss of MTFR1L leads to a decrease in AMPK activation not only at steady state but also upon ETC-induced AMPK activation. While these defects are not due to decreased ATP production measured by bioluminescence assay, it can be hypothesized that loss of MTFR1L induces a cellular metabolic rewiring mimicking mild cellular stress, which could lead to mitochondrial elongation to ensure cell survival. In addition, during selective conditions of stress, including ER stress or proapoptotic signals, mitochondria elongate via a process called stress-induced mitochondrial hyperfusion (SIMH), regulated by the SLP-2/OPA1/Mfn1 axis (*62*, *63*). Thus, loss of MTFR1L could trigger metabolic cellular stress, leading to the activation of SIMH to enhance cell survival. Further experiments should be performed to elucidate the mechanism of how MTFR1L loss leads to increased OPA1 levels and to determine whether loss of MTFR1L could lead to mitochondrial hyperfusion via SIMH. In addition, it will also be important to investigate how expression of the gain of function of MTFR1L or the phosphomimetic mutant MTFR1L^S103D/S238D^ expression induces mitochondrial fragmentation and their perinuclear clustering.

Using biochemical approaches, we confirm MTFR1L as a physiological substrate of AMPK, which is phosphorylated at Ser^103^ and Ser^238^ both under basal conditions and upon AMPK activation. These results confirm previous independent studies that identified MTFR1L as a potential AMPK substrate through different phosphoproteomic screening approaches (*25*, *30*, *31*). We show that MTFR1L is required for AMPK-induced mitochondrial fragmentation, and that these phosphorylation events are critical for the regulation of mitochondrial morphology. MTFR1L^WT^ and MTFR1L^S103D/S238D^ stable overexpression rescued mitochondrial hyperfusion and increased OPA1 protein levels observed in both MTFR1L KO and AMPK-α1α2 DKO cells, respectively, compared to MTFR1L^S103A/S238A^. To date, phosphorylation of ARMC10 and MFF has been associated with regulating mitochondrial fission upon metabolic alterations (*29*, *30*). While ARMC10 interacts with the fission/fusion machinery, its mechanistic role has not yet been elucidated, unlike MFF phosphorylation that has been shown to increase Drp1 recruitment to mitochondria. In contrast, MTFR1L exerts a negative effect on fusion via the modulation of OPA1 levels. Recent studies have highlighted an AMPK-OPA1 connection required to control mitochondrial morphology and cellular homeostasis in different contexts including T cell memory development and the regulation of the cardiovascular system (*64*–*66*). This further emphasizes the plethora of mechanisms by which AMPK regulates mitochondrial dynamics. In this regard, we hypothesize that, depending on the stimulus modulating AMPK activity, different mitochondrial targets could be activated to control mitochondrial morphology, by specifically regulating either mitochondrial fission or fusion. These different levels of regulation will ensure an adequate mitochondrial network, allowing cells to efficiently adapt to different stresses. Collectively, this highlights the complex mechanisms required to control mitochondrial shape under stress conditions, and future studies may unravel the specificity of the AMPK-substrate axis.

Last, it will be essential to decipher which physiological stimuli activate the AMPK-MTFR1L-OPA1 axis to modulate MTFR1L levels for translational approaches. In this connection, modulation of OPA1 levels (and in particular its overexpression) has been previously shown to be beneficial in models of mitochondrial disease, including resistance to apoptosis and enhancement of ETC functionality (*67*, *68*). Thus, increasing OPA1 levels by targeting or inhibiting MTFR1L may represent a potential unexplored therapeutic target for mitochondrial diseases.

## MATERIALS AND METHODS

### Experimental models

#### Cell lines

Cos-7, HeLa, human embryonic kidney (HEK)–293, and U2OS cells were obtained from the American Type Culture Collection. HeLa, HEK-293, and Cos-7 cells were cultured in high-glucose Dulbecco’s modified Eagle’s medium (DMEM) containing d-glucose (4.5 g/liter), sodium pyruvate (0.1 g/liter), and GlutaMAX supplemented with 2 mM l-glutamine, 1% nonessential amino acids, and final 10% of fetal bovine serum (FBS; Life Technologies, Gibco). U2OS cells were cultured in McCoy’s 5A medium (Life Technologies, Gibco) containing the same composition of supplements. AMPK-α1α2 DKO U2OS cells were generated as described in (*49*). Cells were incubated at 37°C with 5% CO_2_. Cells were tested for mycoplasma contamination using the Lookout Mycoplasma PCR Detection Kit (Sigma-Aldrich).

For neuronal culture, mouse brains were dissected out and the cortex was harvested in Hanks’ balanced salt solution (HBSS) supplemented with Hepes (10 mM, pH 7.4). For cocultures, a single electroporated cortex from each condition was dissociated together in papain (Worthington; 14 U/ml) for 15 min at 37°C. After three washes in HBSS, brain cortices were dissociated in 1 ml of neurobasal medium (Invitrogen) with FBS (2.5%), N2 (1×), and B27 (1×) supplements. Dissociated cultures were plated on 35-mm poly-d-lysine (1 mg/ml; Thermo Fisher Scientific)–coated glass-bottom dishes (MatTek) and incubated at 37°C with 5% CO_2_. After day in vitro (DIV) 5, 0.75 ml of culture medium was removed and replaced with 1 ml of fresh neurobasal medium without FBS every other day until imaged.

#### Treatments

ETC inhibitors, antimycin A (Sigma-Aldrich) and rotenone (Sigma-Aldrich), were used to treat cells at 10 μM and 250 ng/ml, respectively, for indicated times, using dimethyl sulfoxide (DMSO) as a control. CCCP (Sigma-Aldrich) was used at 20 μM for 60 min. For cross-linking experiments, cells were treated with 50 μM bismaleimidohexane in medium and incubated for 30 min at 37°C. Cells were then rapidly washed in cold phosphate-buffered saline (PBS) containing dithiothreitol (DTT; 20 mg/ml) and subsequently lysed for immunoblot analysis. For starvation experiments, cells were treated with DMEM lacking glucose (Life Technologies, Gibco) for 2 hours. For cell death experiments analyzed by immunoblot, cells were treated with 10 μM ABT 737 (Stratech Scientific) and 5 μM actinomycin D (Sigma-Aldrich) for 5 hours. For cytochrome c release analysis by immunofluorescence, cells were treated with 10 μM ABT 737, 5 μM actinomycin D, and 10 μM of the pan-caspase inhibitor ZVAD-FMK (R&D Systems) for 5 hours.

#### Mice

All mice were handled in line with Columbia University’s Institutional Animal Care and Use Committee. Timed-pregnant mice were purchased from Charles River Laboratories and electroporated at E15.5 with random assignment to control or experimental condition. Mouse organs were a gift from P. Chinnery (Department of Clinical Neurosciences, School of Clinical Medicine, University of Cambridge, Cambridge, UK).

### Mutagenesis, siRNA, and plasmid transfections

For siRNA experiments, cells were transfected using Lipofectamine RNAiMAX (Invitrogen) with 20 nM siRNA for 3 days, following the manufacturer’s instructions. Nontargeting (ON-TARGETplus SMARTpool D-0001810-10-20), Mfn1 (ON-TARGETplus SMARTpool, L-010670-01-0005), and Mfn2 (ON-TARGETplus SMARTpool L-012961-00-0005) siRNAs were purchased from Dharmacon. OPA1 siRNA was purchased from Sigma-Aldrich (5′-AAGUUAUCAGUCUGAGCCAGGUU-3′), which was previously described in (*55*). MTFR1L siRNAs were purchased from Integrated DNA Technologies (IDT): hs.Ri.MTFR1L.13.1 (si1) and hs.Ri.MTFR1L.13.2 (si2). For transient overexpression, plasmids were transfected using FuGene HD (Roche) according to the manufacturer’s instructions. To generate plasmids containing MTFR1L, MTFR1L gBLOCK (IDT) (5′-ATATATATGAATTCATGGACTACAAGGACGACGATGACAAGATGTCAGGAATGGAAGCCACTGTGACCATCCCAATCTGGCAAAACAAGCCACATGGGGCTGCTCGAAGTGTAGTAAGAAGAATTGGGACCAACCTACCCTTGAAGCCGTGTGCCCGGGCGTCCTTTGAGACCCTGCCCAACATCTCTGACCTGTGTTTGAGAGATGTGCCCCCAGTCCCTACCCTGGCTGACATCGCCTGGATTGCTGCGGATGAAGAGGAGACATATGCCCGGGTCAGGAGTGATACGCGCCCCCTGAGGCACACCTGGAAACCCAGCCCTCTGATTGTCATGCAGCGCAATGCCTCTGTTCCCAACCTGCGTGGGTCCGAGGAGAGGCTTCTGGCCCTGAAGAAGCCAGCTCTGCCAGCCCTAAGCCGCACTACTGAGCTGCAGGACGAGCTGAGCCACTTGCGCAGCCAGATTGCAAAGATAGTGGCAGCTGATGCAGCTTCGGCTTCATTAACGCCAGATTTCTTATCTCCAGGAAGTTCAAATGTCTCTTCTCCCTTACCTTGTTTTGGATCCTCATTCCACTCTACAACTTCCTTTGTCATTAGTGACATCACCGAGGAGACAGAGGTGGAGGTCCCTGAGCTTCCATCAGTCCCCCTGCTTTGTTCTGCCAGCCCTGAATGTTGCAAACCAGAACACAAAGCTGCCTGCAGTTCGTCTGAAGAGGATGACTGCGTCTCTTTGTCCAAGGCCAGCAGCTTTGCAGACATGATGGGTATCCTGAAGGACTTTCACCGAATGAAACAGAGTCAAGATCTGAACCGGAGTTTATTGAAGGAGGAAGACCCTGCTGTGCTTATCTCTGAGGTCCTAAGGAGGAAGTTTGCTCTAAAGGAAGAAGATATCAGTAGAAAAGGAAATTGACTCGAGATATATAT-3′) was digested and ligated into pcDNA 3.1 (Invitrogen) using Eco RI and Xho I restriction enzymes and was subsequently cloned into PCS2+ without a FLAG tag, and into P2A-mCherry-N1 (Addgene, #84329) using Xho I and Age I restriction enzymes. MTFR1L^Δ1–28^ mutant was cloned into P2A-mCherry-N1 using an InFusion HD Cloning kit. MTFR1L phosphorylation site mutants (S103D/S238D; S103A/S238A) were generated using a QuikChange II site-directed mutagenesis kit (Agilent) and cloned into P2A-mCherry-N1.

To express MTFR1L in mouse neurons, sequences were changed from cytomegalovirus (CMV) promoter to pCAG. MTFR1L^WT^ was digested with Eco RI and Xho I and ligated into pcDNA3.1/Puro-CAG backbone from which VSFP-CR was extracted at the same restriction sites (Addgene, #40257) using an InFusion HD Cloning kit. The same backbone was used for the MTFR1L^S103A/S238A^ construct; however, the Nhe I and Not I restriction sites were used.

### Antibodies

Mouse monoclonal anti-VDAC (ab14734), anti-SDHA (ab14715), anti-ATP5a (ab14748), anti-MTCO2 (ab110258), anti-Mic60 (ab110329), and anti-GRP75 (ab2799) antibodies were obtained from Abcam. Rabbit polyclonal anti-Mid49 (16413-A-AP), anti-Mid51 (20164-A-AP), anti-GRP75 (14887-1-AP), anti-MDH2 (15462-1AP), and anti-MFF (17090-A-AP) were obtained from Proteintech. Rabbit polyclonal anti-ACC (3662), anti-AMPK (2532S), anti-pACC (3661), anti-pAMPK (2535), anti-Mfn1 (14739), anti–cleaved caspase 3 (9661), and anti-Mfn2 (11925) were purchased from Cell Signaling Technologies. Mouse monoclonal anti-TOM20 (sc-17764), anti-HSP60 (sc-136291), anti-vinculin (V4505), and anti–α-tubulin (sc-23948) were from Santa Cruz Biotechnology. Mouse monoclonal anti-FLAG (F1804), anti–β-actin (A5541), and rabbit polyclonal MTFR1L (HPA027124) were purchased from Sigma-Aldrich. Mouse monoclonal anti–cytochrome c (556433) was purchased from BD Pharmingen. Mouse monoclonal anti-OPA1 (612607) and anti-Drp1 (611113) were purchased from BD Biosciences. Phospho-MTFR1L antibodies raised against residues Ser^103^ and Ser^238^ were generated from Orygen Antibodies Ltd. For immunoblot analysis, Amersham mouse (NA931)/rabbit (NA934) immunoglobulin G (IgG) horseradish peroxidase–linked from sheep/donkey (GE Healthcare), LICOR IRDye 680RD Goat anti-Rabbit IgG (926-68071) and RDye 800CW Goat anti-Mouse (926-32210) secondary antibodies (LI-COR), and DyLight 800 (Thermo Fisher Scientific) were used. For immunofluorescence, donkey anti-mouse Alexa Fluor 488 (A-21202), goat anti-rabbit Alexa Fluor 594 conjugate (A-11012), and goat anti-mouse highly cross-adsorbed Alexa Fluor Plus 488 (A-32723) secondary antibodies were obtained from Invitrogen.

### Plasmid constructs and oligonucleotides

POCT-PA-GFP and MAPL-FLAG were obtained from H. McBride (McGill University, Canada). P2A-mCherry (84329), TOM20-mCherry (55146), and GFP-OMP25 (141150) were obtained from Addgene. Lentiviral cloning vectors, pWXLd-Ires-Hygro, psPAX2, and pMD2.G were obtained from M. Zeviani (MRC Mitochondrial Biology Unit, Cambridge). Myc-DDK–tagged MTFR1L (RC200212) was obtained from OriGene. The following constructs were already described: pCAG-TdTomato (*69*), pSCV2 (pCAG-mVenus) (*70*), pCAG-mtDsRed (*71*), pCAG-mTAGBFP2 (*54*), pCAG-mtYFP (*69*), pCAG-mtmTAGBFP2 (*69*), pCAG-ActA-mCherry (*72*), and pcDNA3.1/Puro-CAG-VSFP-CR (*73*).

### Subcellular fractionation

Cells were washed once with cold PBS and scraped in mitochondrial isolation buffer (MIB) buffer [220 mM mannitol, 70 mM sucrose, 10 mM Hepes (pH 7.5), 1 mM EGTA, 1× protease cocktail inhibitor, 0.22 μm filtered at pH 7.4]. Cells were broken using a manual 7-ml dounce homogenizer (Kimble chase), at least 80 strokes, on ice, and cell breakage efficiency was checked under the microscope. Whole-cell fractions were saved, followed by centrifugations for 10 min at 800*g*, until no more nuclei and debris were observed. The supernatant was then centrifuged at 9000*g* for 10 min to obtain heavy membrane pellet, containing crude mitochondria [crude mitochondrial fraction (CMF)]. CMF was then washed twice with MIB and recentrifuged at 9000*g* for 15 min. The remaining supernatant of the CMF, containing cytosol and microsomes, was ultracentrifuged at 100,000*g* for 60 min (Beckman Coulter) to separate microsomes (pellet) and cytosolic (supernatant) fractions. All fractions were resuspended in MIB containing 1% Triton X-100 (Sigma-Aldrich). All centrifugation steps were carried out at 4°C.

### Mitochondrial localization assays

Proteinase K protection assay was used to investigate MTFR1L mitochondrial subcompartment localization. Crude mitochondrial pellets obtained, as previously described, were resuspended in MIB and quantified using Bradford assay (Bio-Rad). Proteinase K in modified MIB containing no protease inhibitor [10 mM Hepes (pH 7.4), 68 mM sucrose, 80 mM KCl, 0.5 mM EDTA, 2 mM Mg(CH_3_COO)_2_, 0.22 μm filtered] was used to resuspend 20 μg of mitochondria. Samples were incubated on ice for 30 min, followed by addition of final 2 mM phenylmethylsulfonyl fluoride resuspended in isopropanol on ice to inhibit protease activity. Samples were centrifuged at 10,600*g* for 15 min and washed twice with MIB containing protease inhibitors. Mitochondrial pellets were resuspended in MIB containing protease inhibitors to obtain a final concentration of 1 μg/μl, and the supernatant was discarded. All centrifugations were carried out at 4°C.

To establish whether proteins were inserted into membranes, 30 μg of CMF was treated with 0.1 M sodium carbonate (pH 11) and incubated on ice for 30 min. Samples were ultracentrifuged at 100,000*g* at 4°C for 60 min. The supernatant was saved as fraction containing solubilized proteins, whereas pellet contained inserted proteins. The samples were lysed in final 1% Triton X-100/MIB for 15 min on ice.

### Respirometry

Oxygen consumption measurements were performed in intact cells resuspended in culture DMEM using the Oroboros Instruments High-Resolution Respirometer (*74*). Approximately 3 × 10^6^ cells were used in each experiment. Basal (ROUTINE) respiration was recorded until the steady state was reached. The nonphosphorylating respiration (LEAK) was measured by adding 2.5 μM oligomycin to the chambers to inhibit the ATP synthase, and the respiration rates were left to reach the steady state. The uncoupled state or maximal capacity of the electron transfer system (ETS capacity) was achieved by titrating CCCP in 0.5 μM steps until the respiratory rates did not increase any further. Last, 2.5 μM antimycin A and 1 μM rotenone were added to inhibit complex III and complex I, respectively.

### Immunofluorescence and confocal imaging

Cells were fixed in 5% paraformaldehyde (PFA) in PBS (pH 7.4) for 15 min at 37°C, with three subsequent washes in PBS. Autofluorescence was quenched with 50 mM ammonium chloride for 10 min at room temperature (RT) and three additional washes in PBS. Cells were then permeabilized with Triton X-100/0.1% PBS for 10 min, followed by three washes in PBS. Blocking was carried out in 10% FBS/PBS for 20 min. Primary antibodies were prepared in 5% FBS/PBS and incubated on a shaker for 2 hours at RT or overnight at 4°C. Cells were washed three times in 5% FBS/PBS and incubated with the corresponding secondary antibody (dilution 1: 1000) prepared in 5% FBS/PBS. Coverslips were washed three times in PBS and then in deionized water (DIW), dried, and mounted onto slides using fluorescence mounting medium (Dako).

Images were acquired on a Nikon Eclipse TiE inverted microscope coupled with an Andor Dragonfly 500 confocal spinning disk system using the 60× or 100× objective. Images were captured with seven stacks each of 0.2 μm using the Zyla 4.2 PLUS sCMOS camera (Andor), which was coupled to the Fusion software (Andor). Images were acquired in the same conditions with the same laser intensity (laser lines 488, 568, and 647 nm) and exposure time. Photoactivation of the ROI (25 μm^2^) was performed using the Andor FRAPPA module. Raw seven-stack images were compiled into “max projection,” converted into composites, and proceeded using the Fiji software (*14*).

All neuronal imaging was acquired with a Nikon Ti-E microscope equipped with an A1 confocal. Nikon Elements software was used to control the equipment and lasers. The Nikon 40× oil (1.30 numerical aperture) objective was used for both in vivo and in vitro imaging.

### Transmission electron microscopy

WT and MTFR1L KO cells were seeded at 80 to 90% confluency on Thistle Scientific μ-Dish 35 mm, high, containing Ibidi polymer coverslips (Ibidi, 81151). Cells were washed once with 0.9% saline solution, followed by a 4°C overnight fixation, and were processed for TEM preparation. Samples were fixed in fixative buffer [2% glutaraldehyde/2% formaldehyde in 0.05 M sodium cacodylate buffer (pH 7.4) containing 2 mM calcium chloride] overnight at 4°C. After five washes in 0.05 M sodium cacodylate buffer (pH 7.4), samples were osmicated (1% osmium tetroxide, 1.5% potassium ferricyanide, 0.05 M sodium cacodylate buffer, pH 7.4) for 3 days at 4°C. After five washes in DIW, samples were treated with 0.1% (w/v) thiocarbohydrazide/DIW for 20 min at RT in the dark. After five washes in DIW, samples were osmicated a second time for 60 min at RT (2% osmium tetroxide/DIW). Following five washes in DIW, samples were block-stained with uranyl acetate (2% uranyl acetate in 0.05 M maleate buffer, pH 5.5) for 3 days at 4°C. Samples were washed five times in DIW and then dehydrated in a graded series of ethanol (50%/70%/95%/100%/100% dry) and 100% dry acetonitrile, three times each for at least 5 min. Samples were infiltrated with a 50:50 mixture of 100% dry acetonitrile/Quetol resin [without benzyl dimethylamine (BDMA)] overnight, followed by 3 days in 100% Quetol (without BDMA). Then, the sample was infiltrated for 5 days in 100% Quetol resin with BDMA, exchanging the resin each day. The Quetol resin mixture is 12 g of Quetol 651, 15.7 g of nonenyl succinic anhydride, 5.7 g of methyl nadic anhydride, and 0.5 g of BDMA (all from TAAB). The Ibidi dishes were filled with resin to the rim, covered with a sheet of Aclar, and cured at 60°C for 3 days.

After curing, the Aclar sheets were removed and small sample blocks were cut from the Ibidi dish using a hacksaw. Thin sections (~70 nm) were prepared using an ultramicrotome (Leica Ultracut E). Resin blocks were orientated with the cell side toward the knife, and sections were collected on bare 300-mesh copper grids immediately when reaching the cell monolayer. Samples were imaged in a Tecnai G2 TEM (FEI/Thermo Fisher Scientific) run at 200 keV using a 20-μm objective aperture to improve contrast. Images were acquired using an ORCA HR high-resolution charge-coupled device camera (Advanced Microscopy Techniques Corp., Danvers, USA).

For mitochondrial cristae width analysis, the “measure” function in Fiji ImageJ was used. For cristae analysis, only mitochondria harboring at least five cristae structures clearly visible were analyzed.

### ATP/ADP nucleotide quantification

ATP and ADP concentrations were measured using a luciferase-based assay as described previously (*75*). In brief, treated cells were scraped down into Eppendorf tubes, kept on ice, and lysed in ice-cold perchloric acid extractant [3% (v/v) HClO_4_, 2 mM Na_2_-EDTA, 0.5% Triton X-100] at a concentration of 300 μl/10^5^ cells by vigorously vortexing before transferring samples back onto wet ice. Samples, along with ATP and ADP standards, were neutralized to pH 7 using potassium hydroxide solution (2 M KOH, 2 mM Na_2_-EDTA, and 50 mM MOPS). To measure ADP concentration, 250 μl of neutralized sample was added to 250 μl of ATP sulfurylase assay buffer [20 mM Na_2_MoO_4_, 5 mM guanosine monophosphate (GMP), and 0.2 U of ATP sulfurylase (New England Biolabs, USA)], in tris-HCl buffer [100 mM tris-HCl and 10 mM MgCl_2_ (pH 8.0)], incubated for 30 min at 30°C to convert the sample’s ATP to adenosine phosphosulfate (APS). ADP samples were then heated at 100°C for 5 min and cooled on ice. Standards (100 μl), samples for ATP measurement (100 μl), or samples for ADP measurement (200 μl) (in duplicate) were then added to 400 μl of tris-acetate (TA) buffer (100 mM tris, 2 mM Na_2_-EDTA, and 50 mM MgCl_2_, pH 7.75, with glacial acetic acid) in luminometer tubes. To measure ADP concentration, 10 μl of pyruvate kinase solution [100 mM phosphoenolpyruvate and 6 U of pyruvate kinase suspension (Sigma-Aldrich, #P1506)] was added to one set of ADP standards and one set of ADP samples and then incubated for 30 min at 25°C in the dark to convert ADP to ATP. The duplicate tube (without addition of pyruvate kinase solution) served as an ADP “blank” value. The samples were then all assayed for ATP content in a Berthold AutoLumat Plus LB953 luminometer by addition of 100 μl of luciferase/luciferin solution {7.5 mM DTT, bovine serum albumin (BSA; 0.4 mg/ml), luciferase (1.92 μg/ml) (Sigma-Aldrich, #L9506), 120 μM d-luciferin (Sigma-Aldrich, #L9504), made in TA buffer [25% (v/v) glycerol]}, delivered via auto-injection, protected from light. Bioluminescence of the ATP- dependent luciferase activity was measured for 45 s after injection, and the data were quantified against standard curves.

### SDS–polyacrylamide gel electrophoresis and immunoblot analysis

Cells were lysed in radioimmunoprecipitation assay buffer [20 mM tris (pH 8.0), 150 mM NaCl, 0.1% SDS, 1% deoxycholic acid, 1% NP-40, and complete protease inhibitor cocktail]. For pAMPK analysis, cells were lysed in 50 mM Hepes, 10% glycerol, 0.1% Triton X-100, 1 mM EDTA (pH 7.4), containing 1× protease cocktail inhibitor. Samples were normalized for protein concentration using a Bio-Rad protein assay (Bio-Rad), resolved by SDS–polyacrylamide gel electrophoresis, and transferred to nitrocellulose membrane (0.2 μm pore size; Bio-Rad) or polyvinylidene difluoride membrane (0.2 μm pore size, GE Healthcare) for the detection of phosphoproteins. Next, membranes were blocked with 5% nonfat milk or 5% BSA in PBS for 60 min at RT. Membranes were incubated with primary antibody at the appropriate dilution (in 2% milk or 2% BSA, in 0.05% Tween 20 in PBS) at 4°C overnight. Membranes were washed in 0.05% Tween 20 in PBS three times for 15 min and incubated with appropriate secondary antibodies (1.3,000 in 2% milk or 2% BSA, in 0.05% Tween 20 in PBS). Membranes were treated with Western Lightning Plus ECL (PerkinElmer) and exposed to chemiluminescence either on films (PROTEC) or on a digital ECL machine (Amersham) for image quantification. Immunoblots are representative of at least three biological replicates.

For Fig. 4A, the lysis buffer was 50 mM tris-HCl (pH 7.2), 150 mM NaCl, 1% Triton X-100, 50 mM NaF, 5 mM NaPPi, 1 mM EDTA, 1 mM EGTA, 1 mM DTT, and 1× protease inhibitor cocktail (EDTA-free) (Roche). Proteins were transferred to nitrocellulose membrane using the iBlot 2 system (Thermo Fisher Scientific). Membranes were treated with SuperSignal Western Blotting enhancer kit (Pierce), according to the manufacturer’s instructions, and blocked for 1 hour in LI-COR Odyssey Intercept blocking buffer (PBS). Membranes were incubated at least overnight with primary antibody in primary antibody diluent (Pierce kit). Detection was performed using secondary antibody coupled to IR 680 or IR 800 dye, and the membranes were scanned using the LICOR Odyssey IR imager. Protein concentrations were determined by Coomassie blue binding with BSA as standard.

### Quantitative reverse transcription polymerase chain reaction

Total RNAs from WT and MTFR1L KO U2OS cells and NT- and MTFR1L-silenced U2OS cells were isolated using an RNeasy kit (Qiagen), and cDNA was synthesized using a High-Capacity cDNA RT kit (Applied Biosystems), following the manufacturer’s instructions. mRNA levels were then quantified using the QuantStudio 3 Real-Time PCR System. The following pairs of primers were used: hOPA1, 5′-GGCTCCTGACACAAAGGAAA-3′ (forward) and 5′-TCCTTCCATGAGGGTCCATT-3′ (reverse), as described by (*76*). Glyceraldehyde-3-phosphate dehydrogenase (GAPDH), 5′-GGTGAAGGTCGGAGTCAACG-3′ (forward) and 5′-GAGGGATCTCGCTCCTGGAAG-3′ (reverse).

### KO cell line generation using CRISPR-Cas9

To generate an MTFR1L CRISPR-Cas9–mediated gene KO, U2OS cells were transfected with ps-CAS9-2A-EGFP plasmid containing the gRNA of interest (targeting either exon 3 or 4). gRNA sequences were designed using the online CRISPR software (https://chopchop.cbu.uib.no) and were selected on the basis of having low off-target effects. The following oligonucleotides were used: for targeting exon 3, 5′-CACCgGTGTGCCCGGGCGTCCTTTG-3′ (forward) and 5′-AAACCAAAGGACGCCCGGGCACACc-3′ (reverse); for targeting exon 4, 5′-CACCgTGACATCGCCTGGATTGCTG-3′ (forward) and 5′-AAACCAGCAATCCAGGCGATGTCAc-3′ (reverse). To generate Drp1 KO in HeLa cells, the following gRNA was used: 5′-CACCgAAATAGCTACGGTGAACCCG-3′ (forward) and 5′-AAACCGGGTTCACCGTAGCTATTTc-3′ (reverse). Forty-eight hours after transfection, cells were washed in PBS, trypsinized, and centrifuged. Cell pellet was resuspended in 300 μl of medium and filtered through 70-μm filter into fresh tubes. Cells were sorted into single cell into 96-well plates via fluorescence-activated cell sorting (FACS) and were incubated at 37°C in complete medium containing 20% FBS. After 2 to 3 weeks, single-cell colonies were observed and transferred into 24-well plates. Upon 80% of confluency, clones were tested for efficient KO by immunoblot and polymerase chain reaction (PCR).

### Lentiviral production and transduction

WT, MTFR1L KO, and AMPK-α1α2 DKO U2OS cells were stably transduced with modified pWPXLd-ires-Hygro lentiviral expression vectors (obtained from M. Zeviani, Mitochondrial Biology Unit, University of Cambridge, UK) with P2A-mCherry alone, MTFR1L^WT^-P2A-mCherry, MTFR1L ^S103D/S238D^-P2A-mCherry, and MTFR1L^S103A/S238A^-P2A-mCherry with an HD Infusion Cloning kit. Lentiviral particles were generated in HEK-293T cells via cotransfection of the target vector together with packaging psPAX2 (Addgene, #12260) and envelope pMD2.G (Addgene, #12259) vectors. Twenty-four hours after transduction, cells were selected for hygromycin resistance.

### In utero electroporation/ex utero electroporation

Cortical layer 2/3 PNs were targeted by injecting endotoxin-free plasmid (2 to 5 mg/ml) and 1% Fast Green (Sigma-Aldrich) mixture into the lateral ventricles of E15.5 embryos using Picospritzer III (Parker). Electroporation was performed with five pulses of 45 V for 50 ms (ECM 830, BTX). Mice were then perfused with 4% PFA and 0.75% glutaraldehyde (both from Electron Microscopy Sciences) on postnatal day (P21). Following overnight incubation in fixation mixture, brains were washed three times in PBS before vibratome sectioning (Leica VT1200) at 125 μm and mounted on slides.

For ex utero electroporation, cortical layer 2/3 PNs were targeted by injecting endotoxin-free plasmid (2 to 5 mg/ml) and 1% Fast Green (Sigma-Aldrich) mixture into the lateral ventricles of E15.5 embryos using Picospritzer III (Parker). Electroporation was conducted with four pulses of 20 V for 100 ms (ECM 830, BTX). Plates were then fixed with 4% PFA and 0.75% on DIV14.

### Quantification and statistical analysis

#### Image analysis

For mitochondrial morphology analysis, the mitochondrial network was primarily defined in control cells and categorized as elongated (highly connected, with a minimum of 10 free ends), fragmented (shorter, disassociated), and tubular (neither connected nor disassociated) mitochondria. Mitochondrial networks from treatments/siRNA/KO conditions were assessed and compared to their corresponding controls. At least 30 cells per condition were counted. To further analyze mitochondrial morphology, 225-μm^2^ mitochondrial ROIs from max projection images were selected in the cell periphery, thresholded manually, and were processed with the “smooth” function twice. The mitochondrial area and number analysis quantification was obtained using the “Analyze Particles” function in Fiji, using 0.1 μm as the maximum length measured. For junction analysis, a 225-μm^2^ peripheral mitochondrial ROI was analyzed using the analyze skeleton function, in Fiji. Mitochondrial length was measured using Mitomapr (*77*).

Quantification of mitochondrial fusion was based on previously described methods (*78*, *79*) with minor modifications. The total area of mitochondria was obtained by thresholding images of TOMM20-mCherry, and the photoactivated area was obtained by thresholding images of PA-GFP. The percentage of mitochondria containing PA-GFP was calculated for each time point. Subsequently, the difference in percentage of PA-GFP–containing mitochondria was calculated by subtracting the percentage of photoactivated mitochondria directly after activation from the percentage of photoactivated mitochondria 5 min later.

For live-cell imaging analysis, time-lapse videos were acquired over the course of 5 min with an image captured every 10 s. OMP25-GFP was used to label the mitochondria. A region of 625 μm^2^ per cell was cropped using Fiji software, and the number of fission and fusion events was manually counted. For neurons, quantification was performed in Fiji ImageJ, using the segmented line tool to measure the length of both axonal segments and mitochondria.

#### Statistical analysis

For the bar graphs of mitochondrial morphology, quantitative reverse transcription PCR analysis, immunoblot quantification, and fission/fusion ratio, errors bars displayed on graphs represent the mean ± SEM or SD from at least three independent experiments. For Fig. 6, box plots represent minimum and maximum values, with the box denoting the 25th, 50th, and 75th percentiles from three independent experiments. For all other graphs, data are represented as scatterplots and errors bars displayed on graphs represent the mean ± SEM or SD from at least three independent experiments. The number of cells analyzed is shown in methods. Statistical significance was analyzed using nested one-way analysis of variance (ANOVA), one-way ANOVA, two-way ANOVA, Mann-Whitney, or unpaired *t* test. All statistical analyses were performed using GraphPad Prism software. *P < 0.05, **P < 0.01, ***P < 0.001, and ****P < 0.0001 were considered significant, and *P* > 0.05 was considered nonsignificant (ns).

Figure 2: (C and L): two-way ANOVA, Tukey’s multiple comparison test; (D to F): nested ordinary one-way ANOVA, Dunnett’s multiple comparisons test; (H): Mann-Whitney test. Figure 3: (G and K): two-way ANOVA, Tukey’s multiple comparisons test. (E): two-tailed unpaired Student’s *t* test. (H, I, L, and M): one-way ANOVA, Tukey’s multiple comparisons test. Figure 4: (C, D, F, G, I, and J): nested one-way ANOVA, Dunnett’s multiple comparisons test. Figure 5: (C and D): one-way ANOVA with Tukey’s multiple comparisons test. (B and F to H): two-way ANOVA with Tukey’s multiple comparisons test. Figure 6: (B to D, F to H, and J to L): nonparametric Kruskal-Wallis test with Dunn’s multiple comparison test.

#### Number of cells analyzed by microscopy

For quantification, the number of cells is presented from three independent experiments, otherwise specified.

Figure 2: 2C: *n* = 130, 127, and 138 cells for WT, KO1, and KO2, respectively. 2D: *n* = 63, 63, and 63 for WT, KO1, and KO2, respectively. 2E: *n* = 58, 57, and 58 cells for WT, KO1, and KO2, respectively. 2F: *n* = 99, 86, and 68 cells for WT, KO1, and KO2, respectively. 2H: *n* = 41 and 40 cells for WT and KO, respectively, from four independent experiments. 2J: *n* = 563 and 619 mitochondria counted for WT and KO, respectively. 2L: *n* = 93, 88, 109, and 102 cells for WT, WT + MTFR1L, MTFR1L KO, and MTFR1L KO + MTFR1L, respectively, from two independent experiments.

Figure S2: S2C: *n* = 160, 147, and 155 cells for siNT, si1, and si2, respectively. S2D: *n* = 64, 64, and 44 cells for siNT, si1, and si2, respectively. S2E: *n* = 64, 64, and 50 cells for siNT, si1, and si2, respectively. S2F: *n* = 53, 55, and 43 cells for siNT, si1, and si2, respectively. S2I: *n* = 194, 177, and 175 cells for siNT, si1, and si2, respectively. S2J: *n* = 63, 63, and 55 cells for siNT, si1, and si2, respectively. S2K: *n* = 63, 63, and 55 cells for siNT, si1, and si2, respectively. S2M: *n* = 563 and 619 total mitochondria counted for WT and KO, respectively. S2O: *n* = 46, 45, 45, and 44 cells for WT, WT + MTFR1L, MTFR1L KO, and MTFR1L KO + MTFR1L, respectively. S2P: *n* = 48, 43, 46, and 44 cells for WT, WT + MTFR1L, MTFR1L KO, and MTFR1L KO + MTFR1L.

Figure S3: S3D: *n* = 82, 69, 78, and 117 cells for WT, WT + MTFR1L^Δ1–28^, MTFR1L KO, and MTFR1L KO + MTFR1L^Δ1–28^, respectively. S3E: 58, 41, 55, and 54 cells for WT, WT + MTFR1L^Δ1–28^, MTFR1L KO, and MTFR1L KO + MTFR1L^Δ1–28^, respectively. S3F: 54, 37, 51, and 54 cells for WT, WT + MTFR1L^Δ1–28^, MTFR1L KO, and MTFR1L KO + MTFR1L^Δ1–28^, respectively. S3G: 54, 37, 51, and 54 cells for WT, WT + MTFR1L^Δ1–28^, MTFR1L KO, and MTFR1L KO + MTFR1L^Δ1–28^, respectively.

Figure S5: S5D: *n* = 105, 120, 108, and 94 cells for WT, WT + CCCP, KO, and KO + CCCP, respectively. S5F: *n* = 80, 79, 95, and 79 cells for siNT, siNT + MAPL-FLAG, siMTFR1L, and siMTFR1L + MAPL-FLAG, respectively.

Figure 3: 3E: *n* = 108 and 127 fission events and 92 and 149 fusion events for WT and MTFR1L KO, respectively, from at least 18 cells. 3G: *n* = 102, 99, 130, and 132 cells for WT, WT + siOPA1, KO, and KO + siOPA1, respectively. 3H, I: *n* = 63, 60, 61, and 63 cells for WT, WT + siOPA1, MTFR1L KO, and MTFR1L KO + siOPA1, respectively. 3K: *n* = 101, 112, 152, 148, and 132 cells for WT + siNT, WT + siMfn1, WT + siMfn2, KO + siNT, KO + siMfn1, and KO + siMfn2, respectively. 3L: *n* = 61, 63, 62, 57, 63, and 62 cells for W + siNT, WT + siMfn1, WT + siMfn2, KO + siNT, KO + siMfn1, and KO + siMfn2, respectively. Figure 3M: n = 61, 63, 62, 54, 63, and 62 cells for WT + siNT, WT + siMfn1, WT + siMfn2, KO + siNT, KO + siMfn1, and KO + siMfn2, respectively.

Figure S7: S7E: *n* = 84 and 79 cells for siNT and siMTFR1L in Drp1 KO cells, respectively.

Figure 4: 4C: *n* = 97, 63, 55, and 82 cells for WT U2OS cells stably expressing empty P2A-mCherry, MTFR1L-P2A-mCherry, MTFR1L^S103D/S238D^-P2A-mCherry, and MTFR1L^S103A/S238A^-P2A-mCherry constructs, respectively. 4D: 97, 63, 55, and 82 cells for WT U2OS cells stably expressing empty P2A-mCherry, MTFR1L-P2A-mCherry, MTFR1L^S103D/S238D^-P2A-mCherry, and MTFR1L^S103A/S238A^-P2A-mCherry constructs, respectively. 4F: *n* = 81, 73, 75, and 76 cells for MTFR1L KO cells stably expressing empty P2A-mCherry, MTFR1L-P2A-mCherry, MTFR1L^S103D/S238D^-P2A-mCherry, and MTFR1L^S103A/S238A^-P2A-mCherry constructs, respectively. 4G: 87, 71, 59, and 77 cells for MTFR1L KO cells stably expressing empty P2A-mCherry, MTFR1L-P2A-mCherry, MTFR1L^S103D/S238D^-P2A-mCherry, and MTFR1L^S103A/S238A^-P2A-mCherry constructs, respectively. 4I: 156, 173, 122, and 88 cells for AMPKDKO cells were counted stably expressing empty P2A-mCherry, MTFR1L-P2A-mCherry, MTFR1L^S103D/S238D^-P2A-mCherry, and MTFR1L^S103A/S238A^-P2A-mCherry constructs, respectively. 4J: 151, 167, 120, and 88 cells for AMPKDKO cells stably expressing empty P2A-mCherry, MTFR1L-P2A-mCherry, MTFR1L^S103D/S238D^-P2A-mCherry, and MTFR1L^S103A/S238A^-P2A-mCherry constructs, respectively.

Figure S8: S8B: *n* = 80 mitochondria for WT and MTFR1L KO cells. S8E: *n* = 64, 130, 110, 98, and 112 cells for WT_DMSO, WT_ABT/ActD, MTFR1L KO_DMSO, MTFR1L KO_ABT/ActD, and MTFR1L KO_ siOPA1_ABT/ActD, respectively.

Figure S9: S9B: *n* = 207, 192, 147, and 165 cells for WT U2OS cells stably expressing empty P2A-mCherry, MTFR1L-P2A-mCherry, MTFR1L^S103D/S238D^-P2A-mCherry, and MTFR1L^S103A/S238A^-P2A-mCherry constructs, respectively. S9D: 238, 272, 186, and 209 cells for MTFR1L KO cells stably expressing empty P2A-mCherry, MTFR1L-P2A-mCherry, MTFR1L^S103D/S238D^-P2A-mCherry, and MTFR1L^S103A/S238A^-P2A-mCherry constructs, respectively. S9F: *n* = 331, 296, 270, and 180 cells for AMPKDKO cells stably expressing empty P2A-mCherry, MTFR1L-P2A-mCherry, MTFR1L^S103D/S238D^-P2A-mCherry, and MTFR1L^S103A/S238A^-P2A-mCherry constructs, respectively.

Figure 5: 5B: *n* = 161, 148, 147, 210, 183, and 142 cells for WT_DMSO, WT_AA, WT_C13, KO_DMSO, KO_AA, and KO_C13, respectively. 5C: *n* = 43, 48, 43, 41, 41, and 38 cells for WT_DMSO, WT_AA, WT_C13, KO_DMSO, KO_AA, and KO_13, respectively. 5D: *n* = 43, 48, 43, 41, 41, and 38 cells for WT_DMSO, WT_AA, WT_C13, KO_DMSO, KO_AA, and KO_C13, respectively.

Figure S10: S10B: *n* = 179, 172, 249, and 167 cells for siNT_DMSO, siNT_AA, siMTFR1L_DMSO, and siMTFR1L_AA, respectively.

Figure S11: S11B: *n* = 186, 182, 171, and 194 cells for siNT_DMSO, siNT_rotenone, siMTFR1L_DMSO, and siMTFR1L_rotenone, respectively.

Figure 6: For cocultures, only one axonal segment was quantified per cell: (B to D) *n*_shNT__ = 22 and *n*_shMTFR1L_ = 24; (F to H) *n*_shNT_ = 26, *n*_shMTFR1L_ = 27, *n*_shMTFR1L_WT_ = 21, and *n*_shMTFR1L_S103A/238A_ = 19. The total number of mitochondria per condition was as follows: (B to D) *n*_shNT_ = 310 and *n*_shMTFR1L_ = 506; (F to H) *n*_shNT_ = 248, *n*_shMTFR1L_ = 254, *n*_shMTFR1L_WT_ = 222, and *n*_shMTFR1L_S103A/238A_ = 282. For slices, 33 axonal segments for shNT were quantified across three animals and 31 axonal segments for shMTFR1L. The total number of mitochondria was as follows: (J to L) *n*_shNT_ = 409 and *n*_shMTFR1L_ = 441.

## Notes

View/request a protocol for this paper from *Bio-protocol*.
