# Supplementary material for: AMPK-dependent phosphorylation of MTFR1L regulates mitochondrial morphology
Source: Sci Adv. 2022 Nov 11;8(45):eabo7956. doi: 10.1126/sciadv.abo7956 (PMC9651865; doi:10.1126/sciadv.abo7956)
Supplement: Supplementary file 1 — Figs. S1 to S13 Table S1 [file sciadv.abo7956_sm.pdf]

Science Advances
AAAS

Supplementary Materials for

AMPK-dependent phosphorylation of MTFR1L regulates mitochondrial morphology

Lisa Tilokani *et al.*

Corresponding author: Julien Prudent, julien.prudent@mrc-mbu.cam.ac.uk

Sci. Adv. **8**, eabo7956 (2022)
DOI: 10.1126/sciadv.abo7956

This PDF file includes:

Figs. S1 to S13
Table S1

Supplementary Figure S1

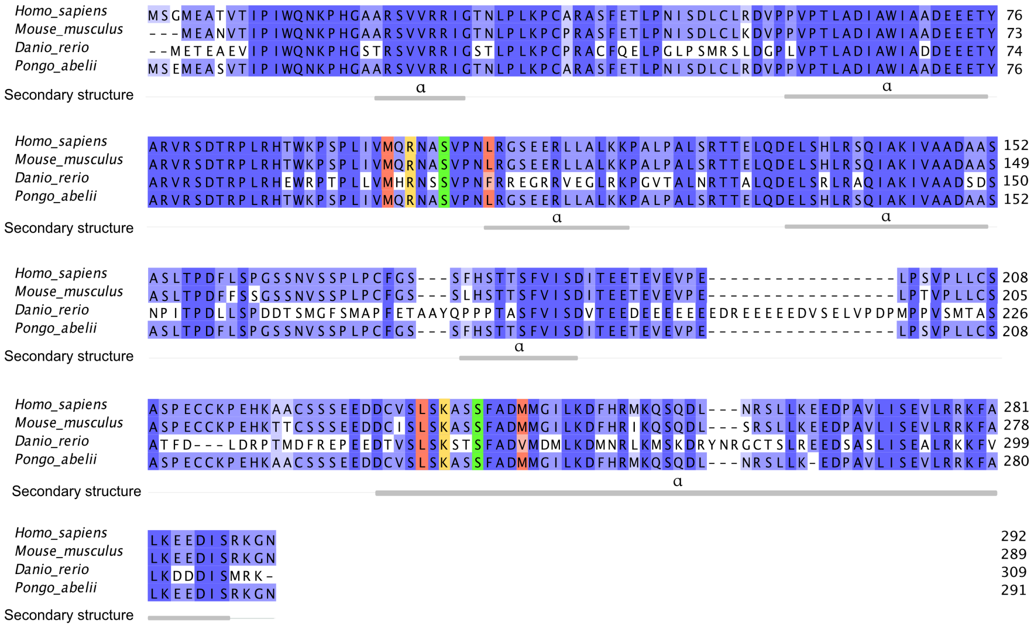

Figure S1, related to Figure 1. MTFR1L protein sequences analysis in vertebrates.

Clustal W multiple sequence alignment carried out in Jalview of MTFR1L from human (NM_19557.5), mouse (Q9CWE0.1), zebrafish (Q0VFP3.1), and monkey (Q5R3Z9.1), together with secondary structure prediction (NPSA Prabi). Conserved phosphorylation sites at Ser103 and Ser238 are highlighted in green. Conserved hydrophobic residues at positions -5 and +4, and basic residues at position -3, relative to the serine phosphorylation sites are highlighted in orange and yellow, respectively.

Supplementary Figure S2

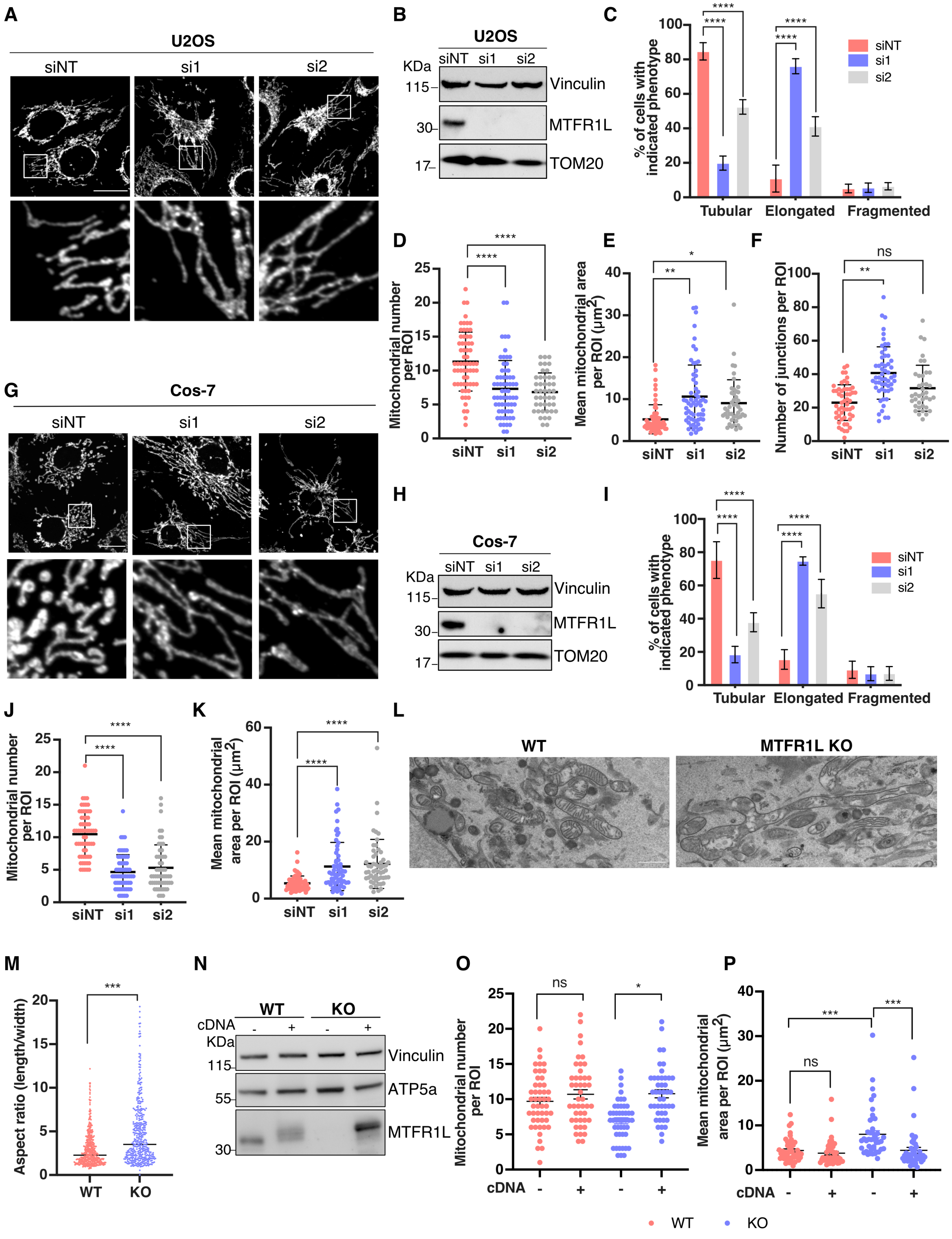

Figure S2, related to Figure 2. Mitochondrial elongation induced by MTFR1L loss is conserved across different cell lines.

(A) Representative confocal images of mitochondrial morphology from U2OS cells treated with non-targeted (NT) or two different MTFR1L small-interference (si) RNAs (si1 and si2). Mitochondria were labelled with an anti-TOM20 antibody. Scale bar, 20 μm.

(B) Immunoblot analysis showing the efficiency of MTFR1L siRNAs in U2OS cells. Vinculin and TOM20 were used as loading controls.

(C) Quantification of mitochondrial morphology from NT and MTFR1L siRNAs-treated U2OS cells from (A). At least 30 cells were counted per experiment; n = 3 independent experiments.

(D-F) Mitochondrial morphology was quantified as: (D) mitochondrial number, (E) mean mitochondrial area, and (F) number of junctions, per region of interest (ROI). At least 15 cells were counted per experiment; n = 3 independent experiments.

(G) Representative confocal images of mitochondrial morphology from Cos-7 cells treated with NT or two different MTFR1L siRNAs (si1 and si2). Mitochondria were labelled with an anti-TOM20 antibody. Scale bar, 20 μm.

(H) Immunoblot analysis showing the efficiency of MTFR1L siRNAs in Cos-7 cells. Vinculin and TOM20 were used as loading controls.

(I) Quantification of mitochondrial morphology from NT and MTFR1L siRNAs-treated Cos-7 cells from (G). At least 30 cells were counted per experiment; n = 3 independent experiments.

(J, K) Mitochondrial morphology was quantified as: (J) mitochondrial number, and (K) mean mitochondrial area, per ROI. At least 15 cells were counted per experiment; n = 3 independent experiments.

(L) Additional examples of transmission electron microscopy (TEM) images from wild-type (WT) and MTFR1L knock-out (KO) U2OS cells. Scale bar, 1 μm.

(M) Quantification of TEM images showing the width / length aspect ratio of mitochondria in WT and MTFR1L KO U2OS cells from Figure 2I. At least 400 mitochondria were counted; n = 2 independent experiments.

(N) Immunoblot analysis showing the efficiency of MTFR1L-P2A-mCherry transient expression (+) in WT and MTFR1L KO U2OS cells. P2A-mCherry alone vector (-) was used as a control. Vinculin and ATP5a were used as loading controls.

(O, P) Mitochondrial morphology was quantified from Figure 2K as (O) mitochondrial number, and (P) mean mitochondrial area, per ROI. At least 15 cells were counted per experiment; n = 3 independent experiments.

All values: mean ± SD; at least three independent experiments. (C, I) Two-way ANOVA, Tukey's multiple comparison test; (D-F; J, K): Nested ordinary one-way ANOVA, Dunnet's multiple comparisons test; (M): non-parametric Mann-Whitney test; (O, P): Nested one-way ANOVA, Tukey's multiple comparisons test. * $p<0.05$; ** $p<0.01$; *** $p<0.001$; **** $P<0.0001$; ns $p>0.05$.

Supplementary Figure S3

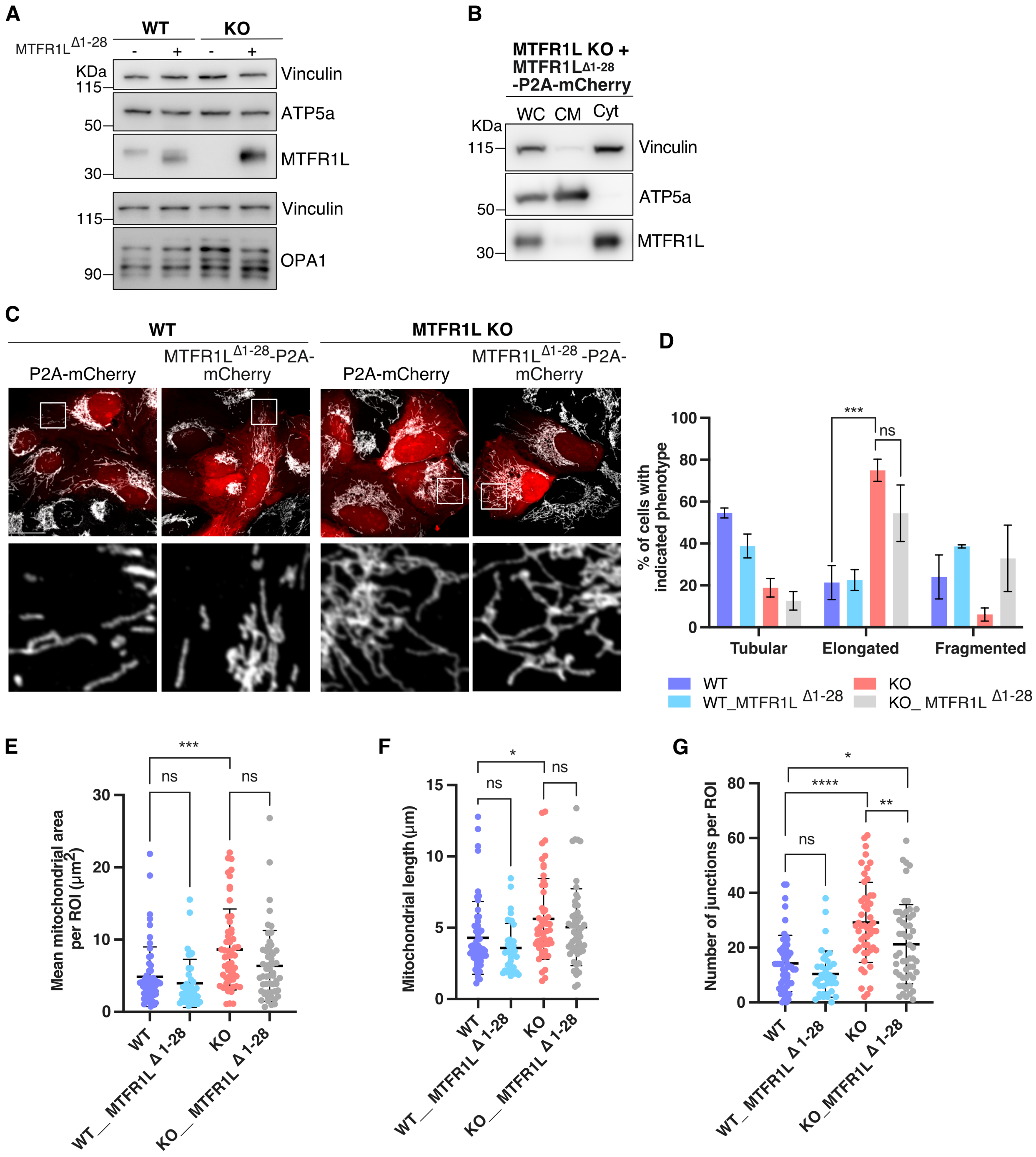

Figure S3, related to Figure 2. The N-terminal domain of MTFR1L is required for regulating mitochondrial morphology.

(A) Immunoblot analysis showing the efficiency of MTFR1L$^{\Delta1\text{-}28}$-P2A-mCherry transient overexpression (+) in wild-type (WT) and MTFR1L knock-out (KO) U2OS cells. P2A-mCherry alone vector (-) was used as a control. Vinculin and ATP5a were used as loading controls. The same lysates were used to analyse OPA1 isoforms levels. Vinculin was used as a loading control.

(B) Subcellular fractionation analysis of MTFR1L$^{\Delta1\text{-}28}$ distribution in MTFR1L KO U2OS cells transiently overexpressing MTFR1L$^{\Delta1\text{-}28}$-P2A-mCherry. Total whole cell (WC) lysates were fractionated into heavy membranes, containing crude mitochondria (CM), and cytosolic (Cyt) fractions. Vinculin and ATP5a were used as cytosolic and mitochondrial markers, respectively.

(C) Representative confocal images of mitochondrial morphology of WT and MTFR1L KO U2OS cells transiently overexpressing P2A-mCherry alone or MTFR1L$^{\Delta1\text{-}28}$-P2A-mCherry. Mitochondria were labelled with an anti-TOM20 antibody. Scale bar, 20 μm.

(D) Quantification of mitochondrial morphology from (C). At least 30 cells were counted per experiment; n = 3 independent experiments.

(E-G) Mitochondrial morphology was quantified as: (D) mean mitochondrial area, (E) mitochondrial length, and (F) number of junctions, per region of interest (ROI). At least 15 cells were counted per experiment; n = 3 independent experiments.

All values: mean ± SD; at least three independent experiments. (D) Two-way ANOVA, Tukey's multiple comparison test; (E-G): ordinary one-way ANOVA, Tukey's multiple comparisons test; * $p<0.05$; ** $p<0.01$; ***$p<0.001$; **** $P<0.0001$; ns $p>0.05$.

Supplementary Figure S4

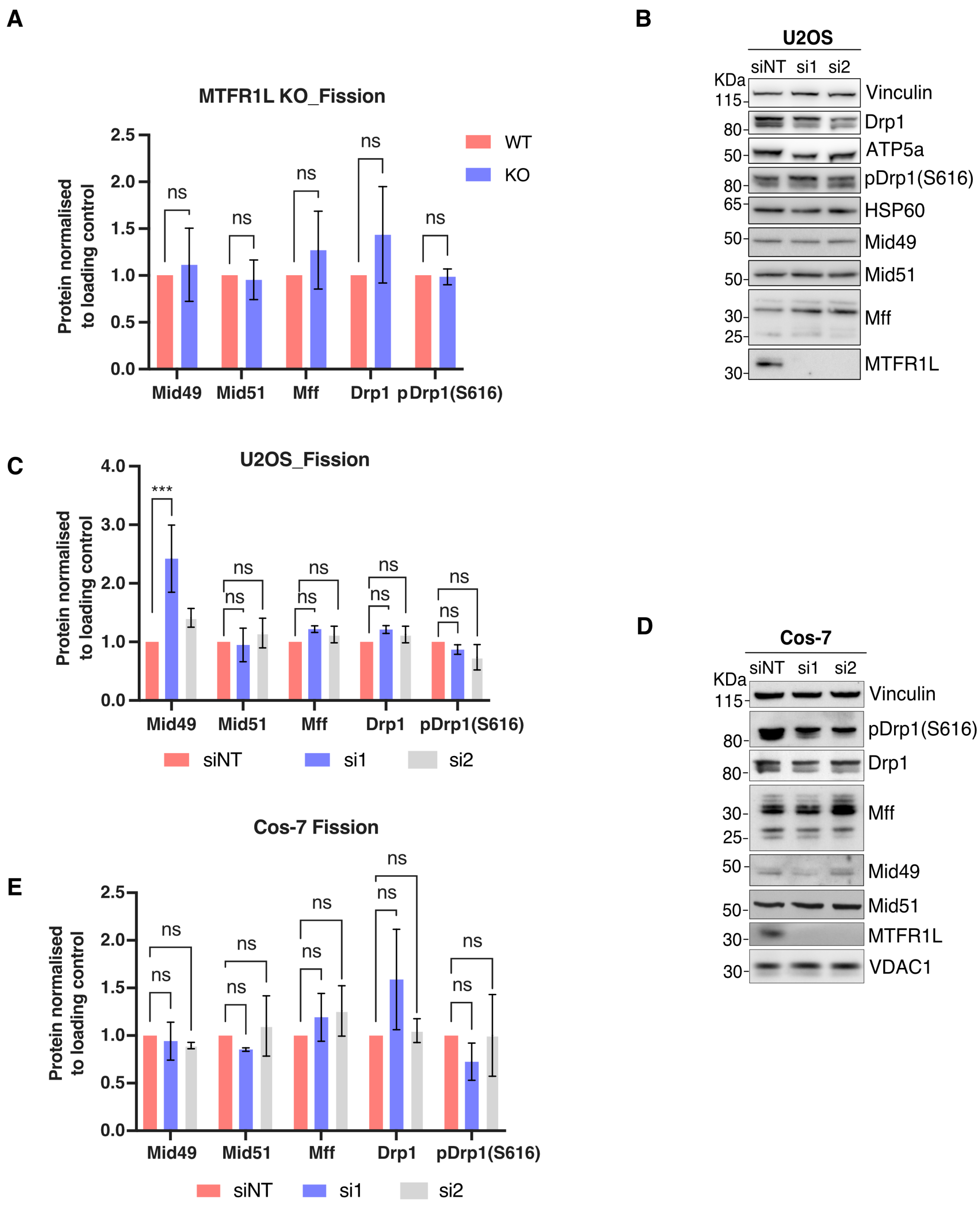

Figure S4, related to Figure 3. Loss of MTFR1L does not affect mitochondrial division and its respective machinery.

(A) Quantification of levels of the indicated proteins from Figure 3A. Signal intensities were quantified by densitometry and normalized to loading controls. N = 3 independent experiments.

(B, D) Immunoblots of proteins related to mitochondrial division from (B) U2OS cells, and (D) Cos-7 cells, treated with non-targeted (NT) or two different MTFR1L small-interference (si) RNAs (si1 and si2). Vinculin, VDAC1, ATP5a, MTCO2 and HSP60 were used as loading controls.

(C, E) Quantification of levels of the indicated proteins from (C) U2OS cells in (B), and (E) Cos-7 cells in (D). Signal intensities were quantified by densitometry and normalized to loading controls. N = 3 independent experiments.

All values: mean ± SEM; at least three independent experiments. (A, C, E): Two-way ANOVA, Tukey's multiple comparisons test, * $p<0.05$; ** $p<0.01$; ***$p<0.001$; **** $P<0.0001$; ns $p>0.05$.

Supplementary Figure S5

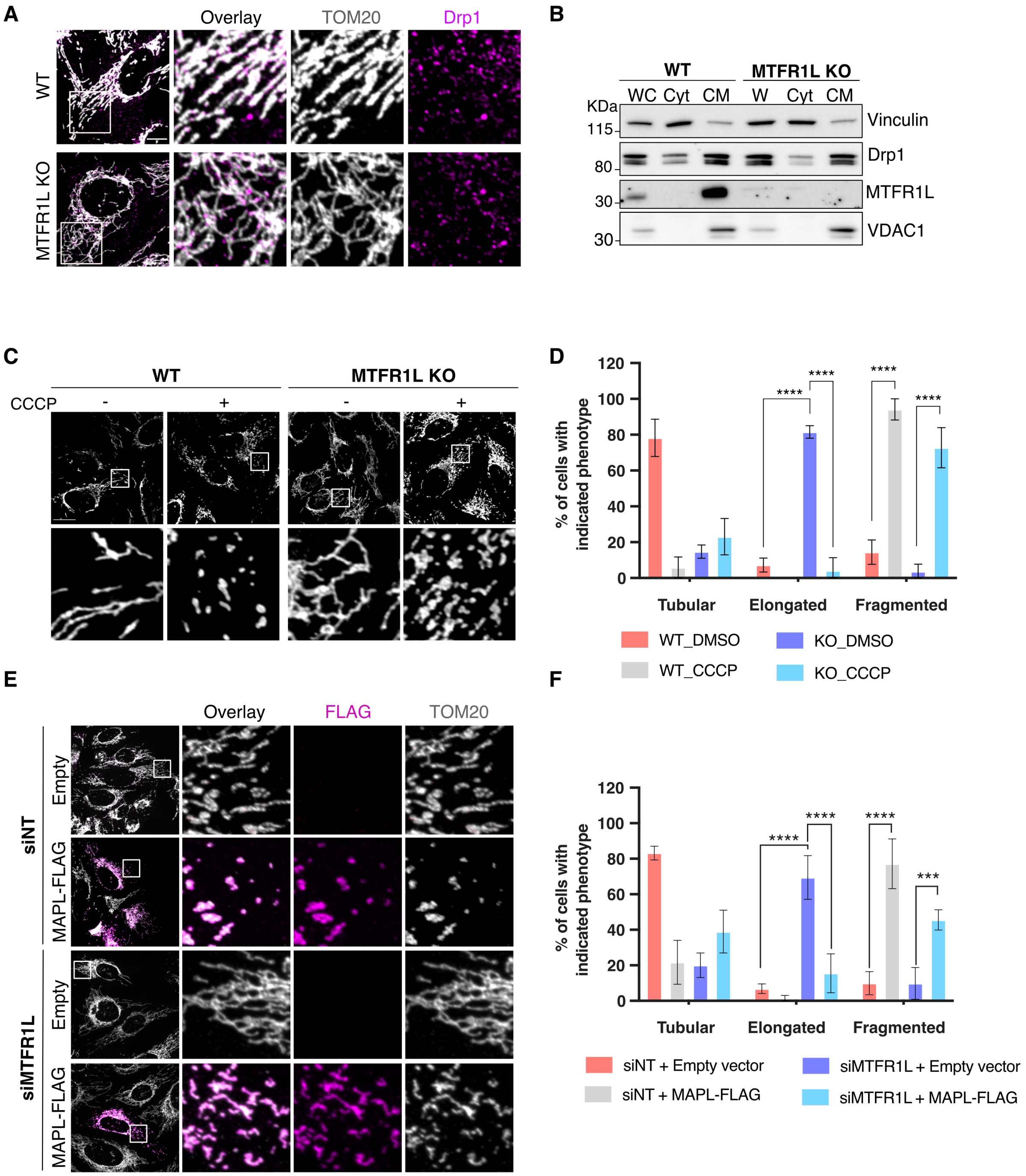

Figure S5, related to Figure 3. MTFR1L KO cells are sensitive to stimuli that trigger Drp1-dependent division.

(A) Representative confocal images of mitochondrial morphology and Drp1 localization in wild-type (WT) and MTFR1L knock-out (KO) U2OS cells. Drp1 and TOM20 were labelled with anti-Drp1 and anti-TOM20 antibodies, respectively. Scale bar, 20 µm.

(B) Subcellular fractionation analysis of Drp1 distribution in WT and MTFR1L KO U2OS cells. Total whole cell (WC) lysates were fractionated into heavy membranes, containing crude mitochondria (CM), and cytosolic (Cyt) fractions. Vinculin and VDAC1 were used as cytosolic and mitochondrial markers, respectively.

(C) Representative confocal images of mitochondrial morphology in WT and MTFR1L KO U2OS cells, treated with DMSO (-) or 20 µM CCCP (+) for 1 hour. Mitochondria were labelled with an anti-TOM20 antibody. Scale bar, 20 µm.

(D) Quantification of mitochondrial morphology from (C). At least 30 cells were counted per experiment; n = 3 independent experiments.

(E) Representative confocal images of mitochondrial morphology in non-targeted (NT) or MTFR1L small-interference (si) RNA treated U2OS cells transiently overexpressing empty vector (pcDNA 3.0) or MAPL-FLAG. Mitochondria and FLAG were labelled with anti-TOM20 and anti-FLAG antibodies, respectively. Scale bar, 20 µm.

(F) Quantification of mitochondrial morphology from (E). At least 30 cells were counted per experiment; n = 3 independent experiments.

All values: mean ± SD; at least three independent experiments; (D, F): Two-way ANOVA, Tukey's multiple comparisons test. * $p<0.05$; ** $p<0.01$; ***$p<0.001$; **** $P<0.0001$; ns $p>0.05$.

Supplementary Figure S6

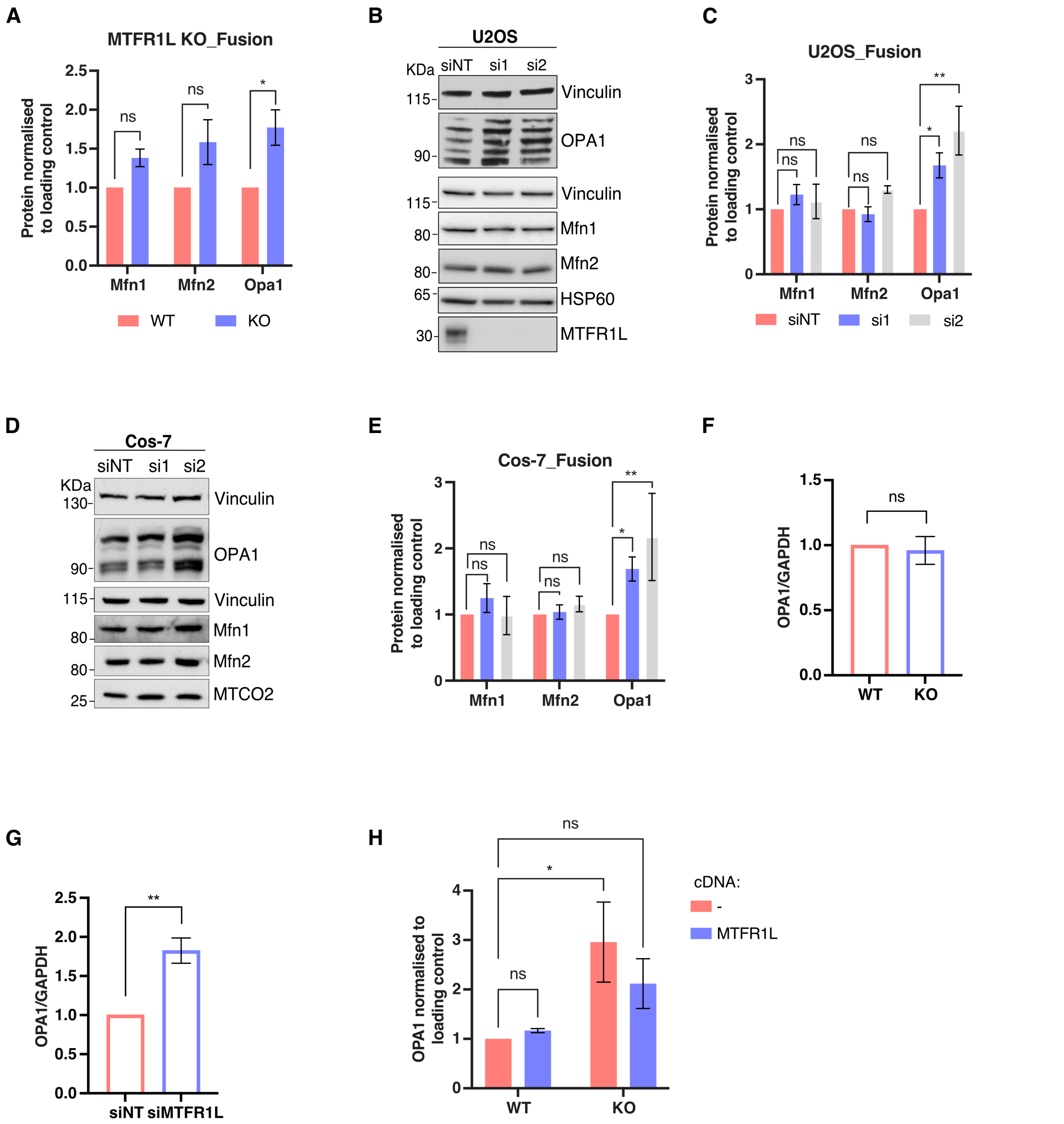

Figure S6, related to Figure 3. Loss of MTFR1L induces increased mitochondrial fusion and OPA1 protein levels.

(A) Quantification of levels of the indicated proteins from Figure 3B. Signal intensities were quantified by densitometry and normalized to loading controls. N = 3 independent experiments.

(B, D) Immunoblots of proteins related to mitochondrial fusion from (B) U2OS cells, and (D) Cos-7 cells, treated with non-targeted (NT) or two different MTFR1L small-interference (si) RNAs (si1 and si2). Vinculin, HSP60 and MTCO2 were used as loading controls.

(C, E) Quantification of levels of the indicated proteins from (C) U2OS cells in (B), and (E) Cos-7 cells in (D). Signal intensities were quantified by densitometry and normalized to loading controls. N = 3 independent experiments.

(F) Quantification of OPA1 mRNA in wild-type (WT) and MTFR1L knock-out (KO) U2OS cells normalised to GAPDH mRNA, and expressed relative to WT control cells. N = 5 independent experiments.

(G) Quantification of OPA1 mRNA in NT- and MTFR1L-silenced U2OS cells normalised to GAPDH mRNA, and expressed relative to siNT-treated U2OS cells. N = 3 independent experiments.

(H) Quantification of total OPA1 levels from Figure 3D. Signal intensities were quantified by densitometry and normalized to loading controls. N = 3 independent experiments.

All values: mean ± SEM; at least three independent experiments. (A, C, E, H): Two-way ANOVA, Tukey's multiple comparisons test. (F, G): two-tailed unpaired student's t-test. * $p<0.05$; ** $p<0.01$; ***$p<0.001$; **** $P<0.0001$; ns $p>0.05$.

Supplementary Figure S7

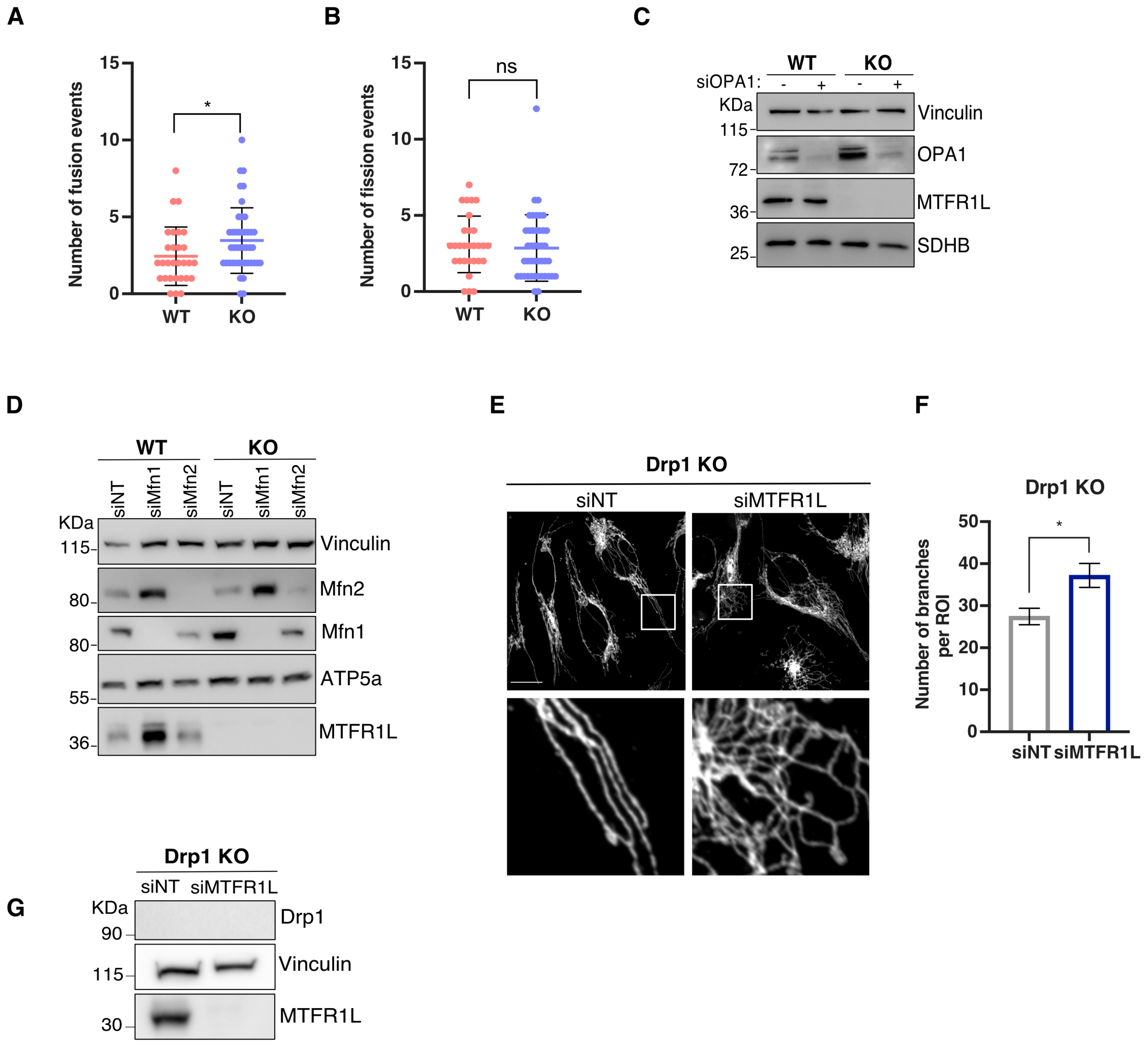

Figure S7, related to Figure 3. Loss of MTFR1L induces increased mitochondrial fusion events.

(A, B) Quantification of (A) mitochondrial fusion events, and (B) fission events, from wild-type (WT) and MTFR1L knock-out (KO) U2OS cells transiently overexpressing the mitochondrial marker, GFP-OMP25. Representative quantification of events per 625 µm^2 region of interest (ROI) over a 5-minutes period. At least 6 cells were counted per experiment; n = 3 independent experiments.

(C) Immunoblot analysis showing the efficiency of OPA1 small-interference (si)RNA in WT and MTFR1L KO U2OS cells. Vinculin and SDHB were used as loading controls.

(D) Immunoblot analysis showing the efficiency of Mfn1 and Mfn2 siRNAs in WT and MTFR1L KO U2OS cells. Vinculin and ATP5a were used as loading controls.

(E) Representative confocal images of mitochondrial morphology from Drp1 KO HeLa cells silenced with NT or MTFR1L siRNAs. Mitochondria were labelled with an anti-TOM20 antibody. Scale bar, 20 μm.

(F) Mitochondrial morphology was quantified from (E) as mitochondrial number of junctions, per region of interest (ROI). At least 15 cells were counted per experiment; n = 3 independent experiments.

(G) Immunoblot analysis showing the efficiency of MTFR1L knockdown at three days in Drp1 KO cells. Vinculin was used as a loading control.

All values: mean ± SD; at least three independent experiments. (A, B): Mann-Whitney test. (F): two-tailed unpaired student's t-test; * $p<0.05$; ** $p<0.01$; *** $p<0.001$; **** $P<0.0001$; ns $p>0.05$.

Supplementary Figure S8

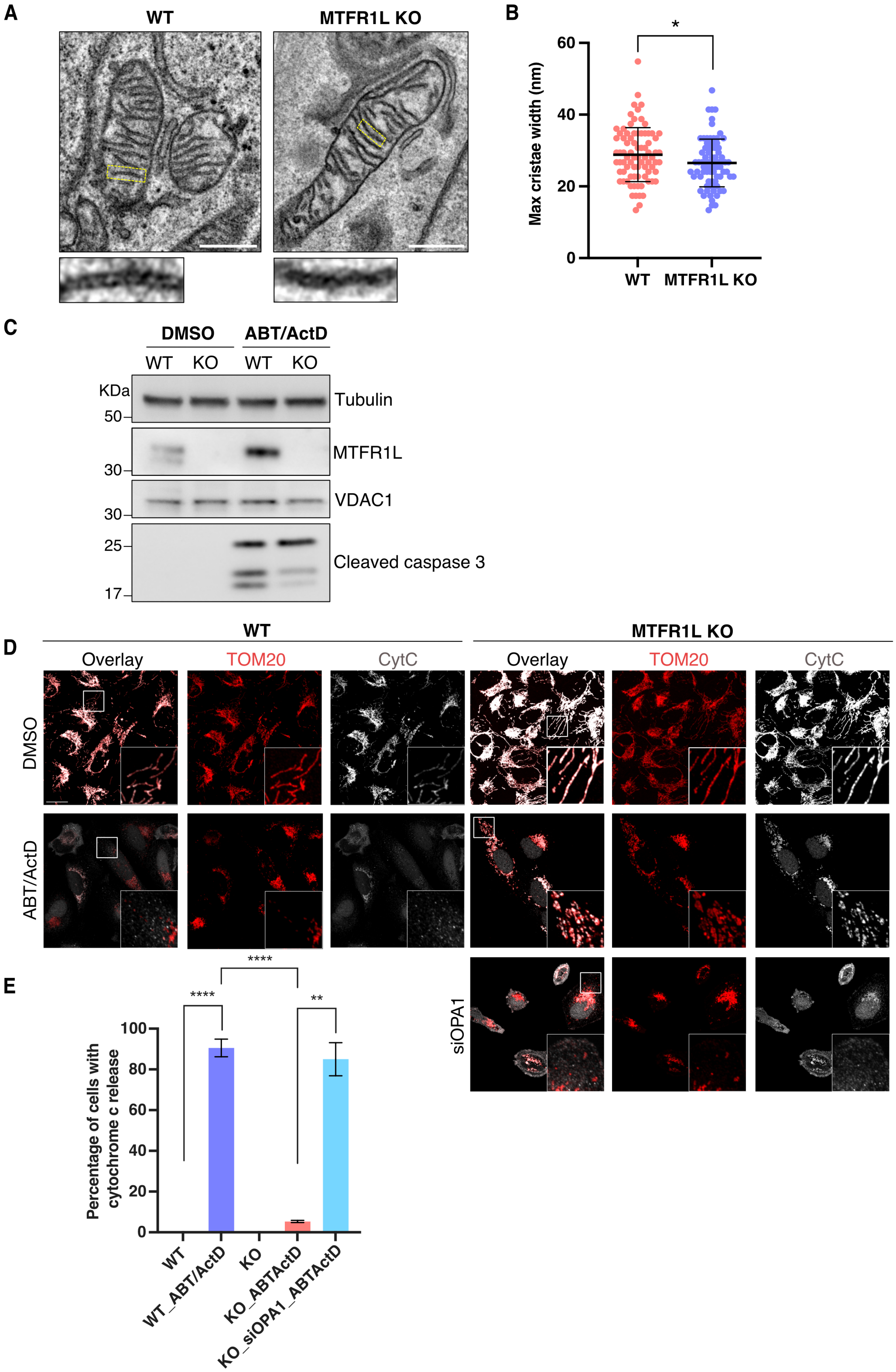

Figure S8. MTFR1L KO cells are resistant to apoptotic insults.

(A) Representative transmission electron micrographs from wild-type (WT) and MTFR1L knock-out (KO) U2OS cells. Scale bar, 500 nm

(B) Quantification of mitochondrial cristae width from WT and MTFR1L U2OS KO cells. At least 40 mitochondria were counted per experiment; n = 2 independent experiments.

(C) Immunoblot analysis of indicated proteins from WT and MTFR1L KO U2OS cells treated with DMSO (-), or with 10 μM ABT 737 and 5 μM Actinomycin D (+) for 5 hours. Tubulin and VDAC1 were used as loading controls.

(D) Representative confocal images from WT and MTFR1L KO U2OS cells silenced with OPA1 small-interference (si)RNA, treated with DMSO, or with 10 μM ABT 737, 5 μM Actinomycin D and 10 μM ZVAD for 5 hours. Mitochondria and cytochrome c were labelled with anti-TOM20 and anti-cytochrome c antibodies, respectively. Scale bar, 20 μm.

(E) Quantification of cells from (D) with cytosolic cytochrome c. At least 15 cells were counted per experiment; n = 3 independent experiments.

All values: mean ± SD; at least three independent experiments. (B): two-tailed unpaired student's t-test; (E): One-way ANOVA, Tukey's multiple comparisons test. * $p<0.05$; ** $p<0.01$; ***$p<0.001$; **** $P<0.0001$; ns $p>0.05$.

Supplementary Figure S9

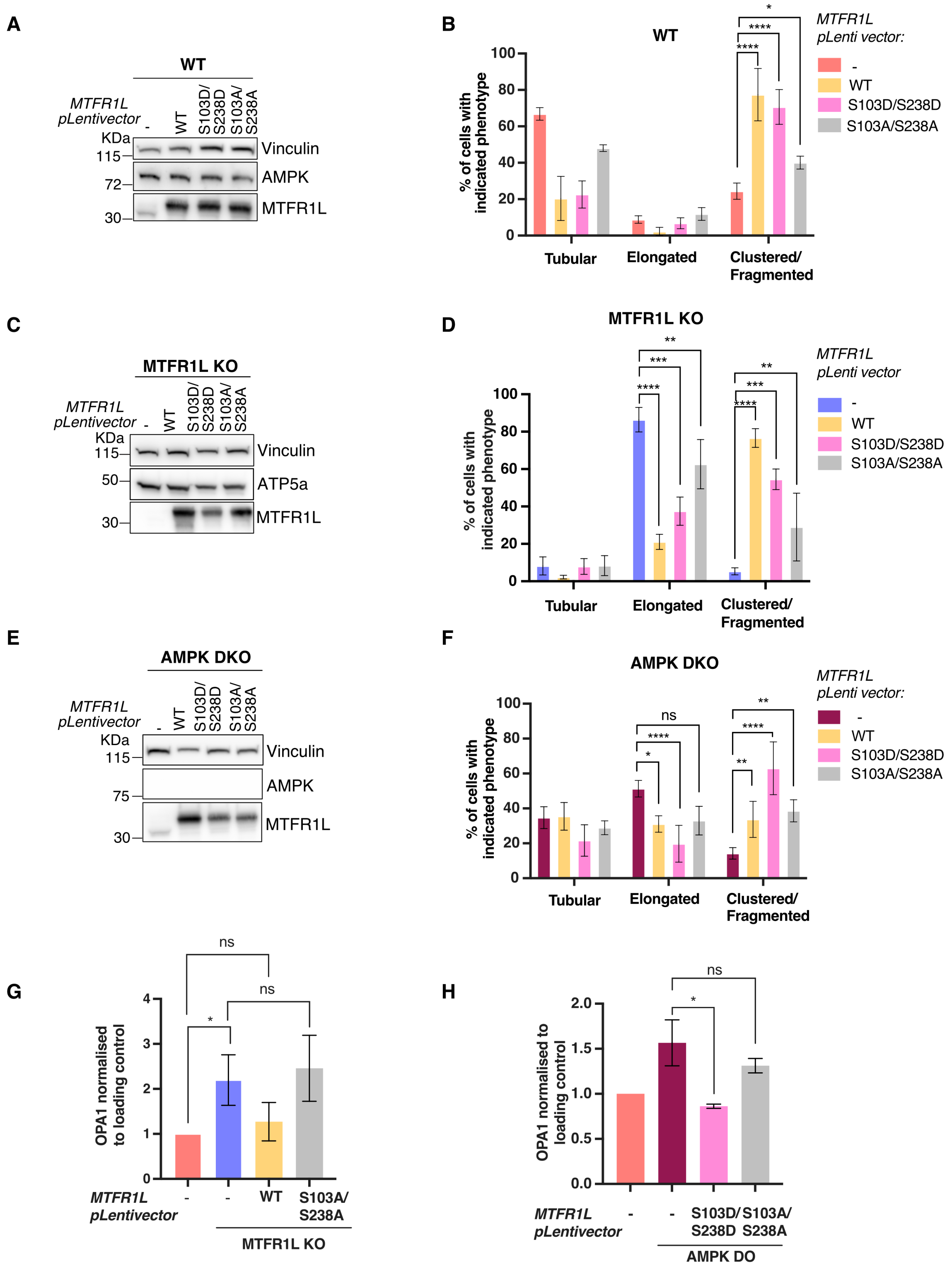

Figure S9, related to Figure 4. AMPK-dependent phosphorylation of MTFR1L controls mitochondrial morphology.

(A, C, E) Immunoblot analysis of indicated proteins showing the efficiency of MTFR1L-P2A-mCherry, MTFR1L$^{S103D/S238D}$-P2A-mCherry, and MTFR1L$^{S103A/S238A}$-P2A-mCherry stable expression in (A) wild-type (WT), (C) MTFR1L knock-out (KO), and (E) AMPK-α1α2 double KO (DKO) U2OS cells. P2A-mCherry alone vector was used as a control. Vinculin and ATP5a were used as loading controls.

(B, D, F) Quantification of mitochondrial morphology from (B) WT (Figure 4B), (D) MTFR1L KO (Figure 4E), and (F) AMPK-α1α2 DKO (Figure 4H) U20S cells stably expressing P2A-mCherry, MTFR1L-P2A-mCherry, MTFR1L-$^{S103D/S238D}$-P2A-mCherry, and MTFR1L-$^{S103A/S238A}$-P2A-mCherry. At least 30 cells were counted per experiment; n = 3 independent experiments.

(G, H) Quantification of OPA1 levels from Figure 4K, L. Signal intensities were quantified by densitometry and normalized to loading controls. N = 3 independent experiments.

Values (B, D, F): mean ± SD; Values (G, H): mean ± SEM; of at least three independent experiments. (B, D, F): Two-way ANOVA, Tukey's multiple comparisons test; (G) Mann-Whitney t-test; (H) Kruskal-Wallis test with Dunn's multiple comparison test * $p<0.05$; ** $p<0.01$; ***$p<0.001$; **** $P<0.0001$; ns $p>0.05$.

Supplementary Figure S10

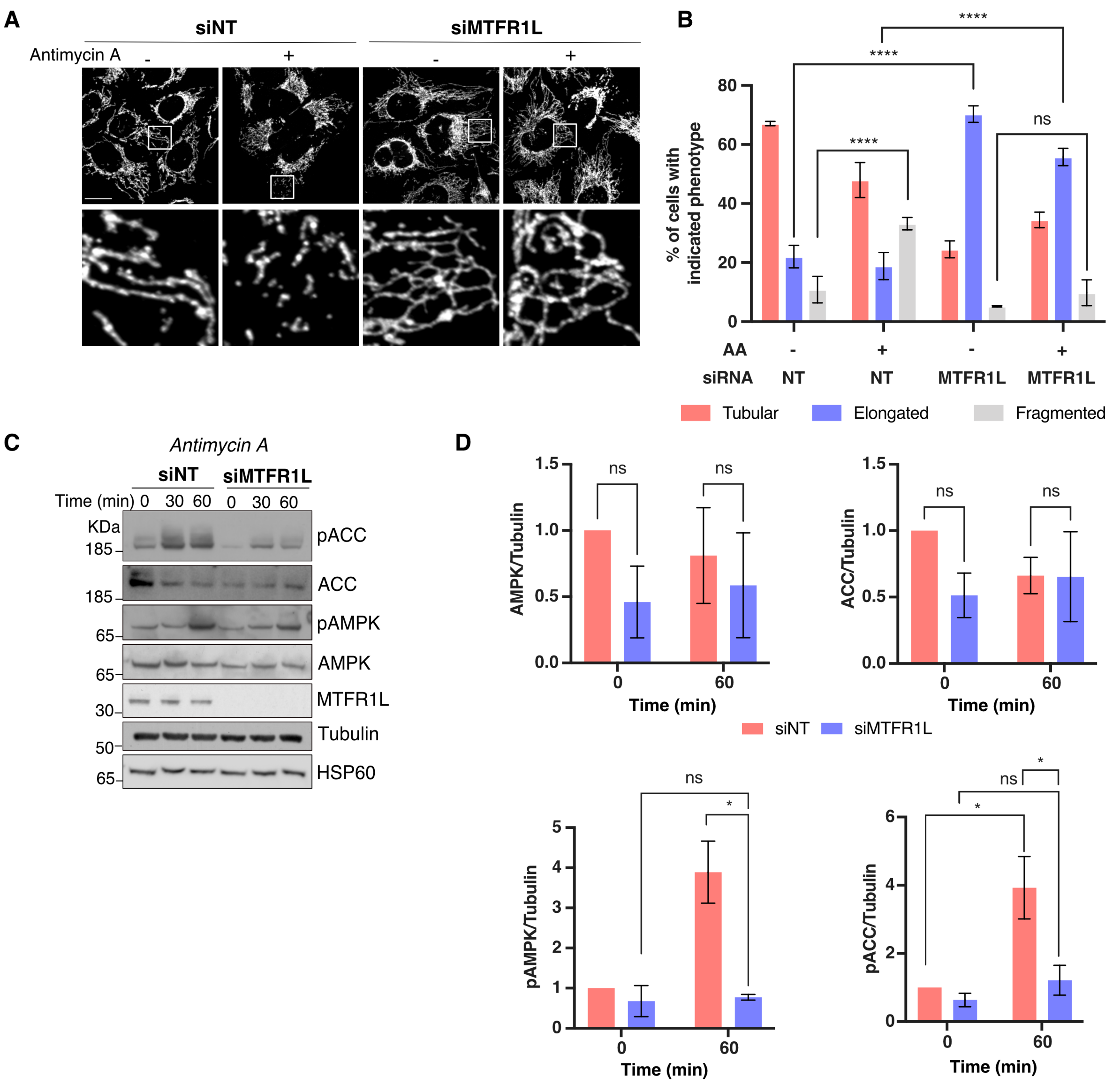

Figure S10, related to Figure 5. MTFR1L silenced cells are resistant to AMPK-induced mitochondrial fragmentation during Antimycin A treatment.

(A) Representative confocal images of mitochondrial morphology from U2OS cells silenced with non-targeted (NT) or MTFR1L small-interference (si) RNAs, and treated with DMSO (-), or 10 µM antimycin (+) A for 1 hour. Mitochondria were labelled with an anti-TOM20 antibody. Scale bar, 20 µm.

(B) Quantification of mitochondrial morphology from (A). At least 30 cells were counted per experiment; n = 3 independent experiments; AA: antimycin A.

(C) Immunoblot analysis of indicated proteins corresponding to (A) showing AMPK activation in U2OS cells silenced with NT or MTFR1L siRNAs, and treated with DMSO, or 10 µM antimycin A for indicated times. Tubulin and HSP60 were used as loading controls.

(D) Quantification of levels of the indicated proteins at 1 hour of treatment from (C). Signal intensities were quantified by densitometry and normalized to loading controls. N = 3 independent experiments.

All values: mean ± SD; at least three independent experiments. (B, D): Two-way ANOVA, Tukey's or Sidak's multiple comparisons test. * $p<0.05$; ** $p<0.01$; ***$p<0.001$; **** $p<0.0001$; ns $p>0.05$.

Supplementary Figure S11

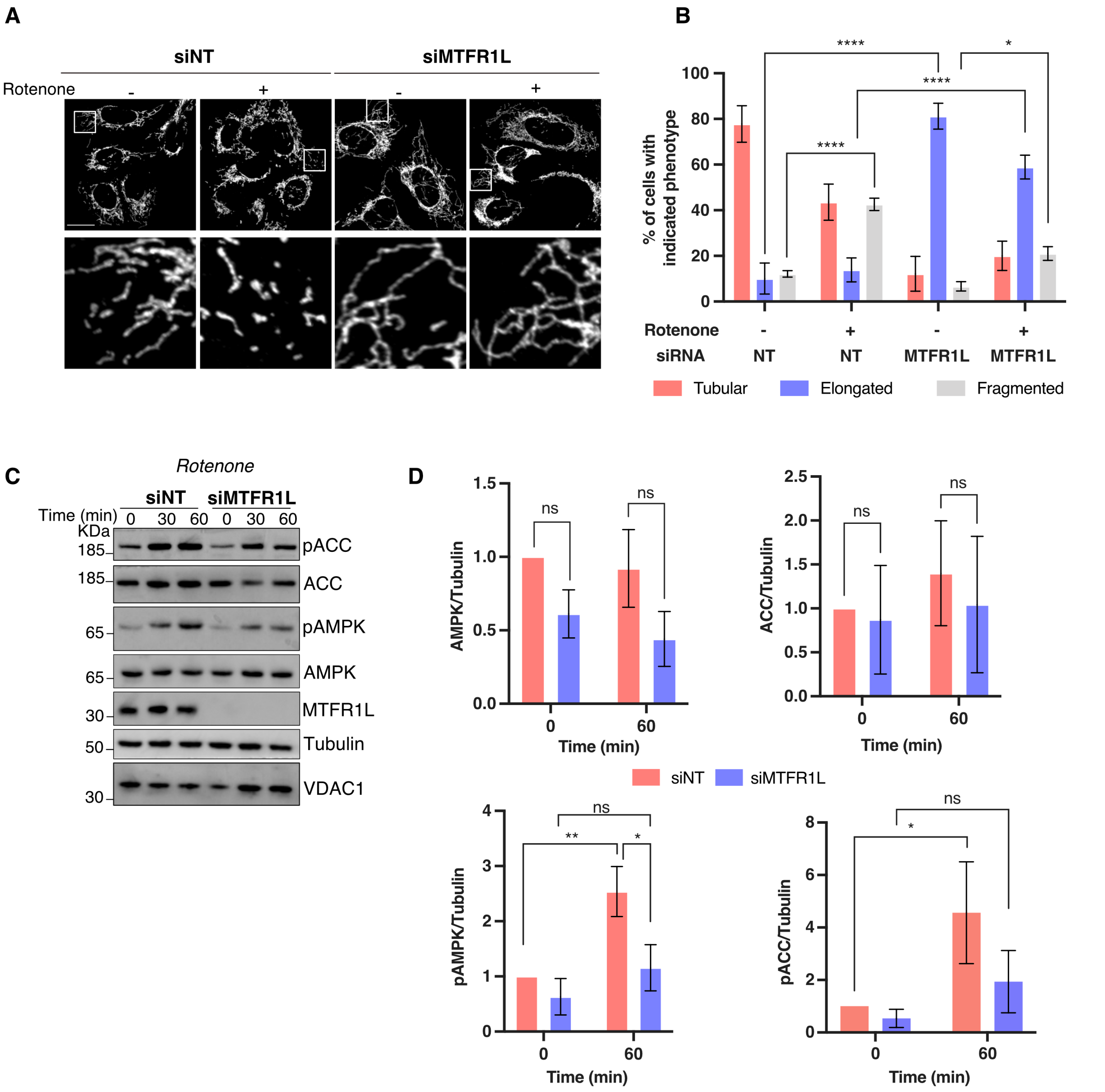

Figure S11, related to Figure 5. MTFR1L silenced cells are resistant to AMPK-induced mitochondrial fragmentation during Rotenone treatment.

(A) Representative confocal images of mitochondrial morphology from U2OS cells silenced with non-targeted (NT) or MTFR1L small-interference (si) RNAs, and treated with DMSO (-), or 250 ng/mL rotenone (+) for 1 hour. Mitochondria were labelled with an anti-TOM20 antibody. Scale bar, 20 µm.

(B) Quantification of mitochondrial morphology from (A). At least 30 cells were counted per experiment; n = 3 independent experiments.

(C) Immunoblot analysis of indicated proteins corresponding to (A) showing AMPK activation in U2OS cells silenced with NT or MTFR1L siRNAs, and treated with DMSO, or 250 ng/mL rotenone for indicated times. Tubulin and VDAC1 were used as loading controls.

(D) Quantification of levels of the indicated proteins from (C). Signal intensities were quantified by densitometry and normalized to loading controls. N = 3 independent experiments.

All values: mean ± SD; at least three independent experiments. (B, D): Two-way ANOVA, Tukey's or Sidak's multiple comparisons test. * $p<0.05$; ** $p<0.01$; ***$p<0.001$; **** $p<0.0001$; ns $p>0.05$.

Supplementary Figure S12

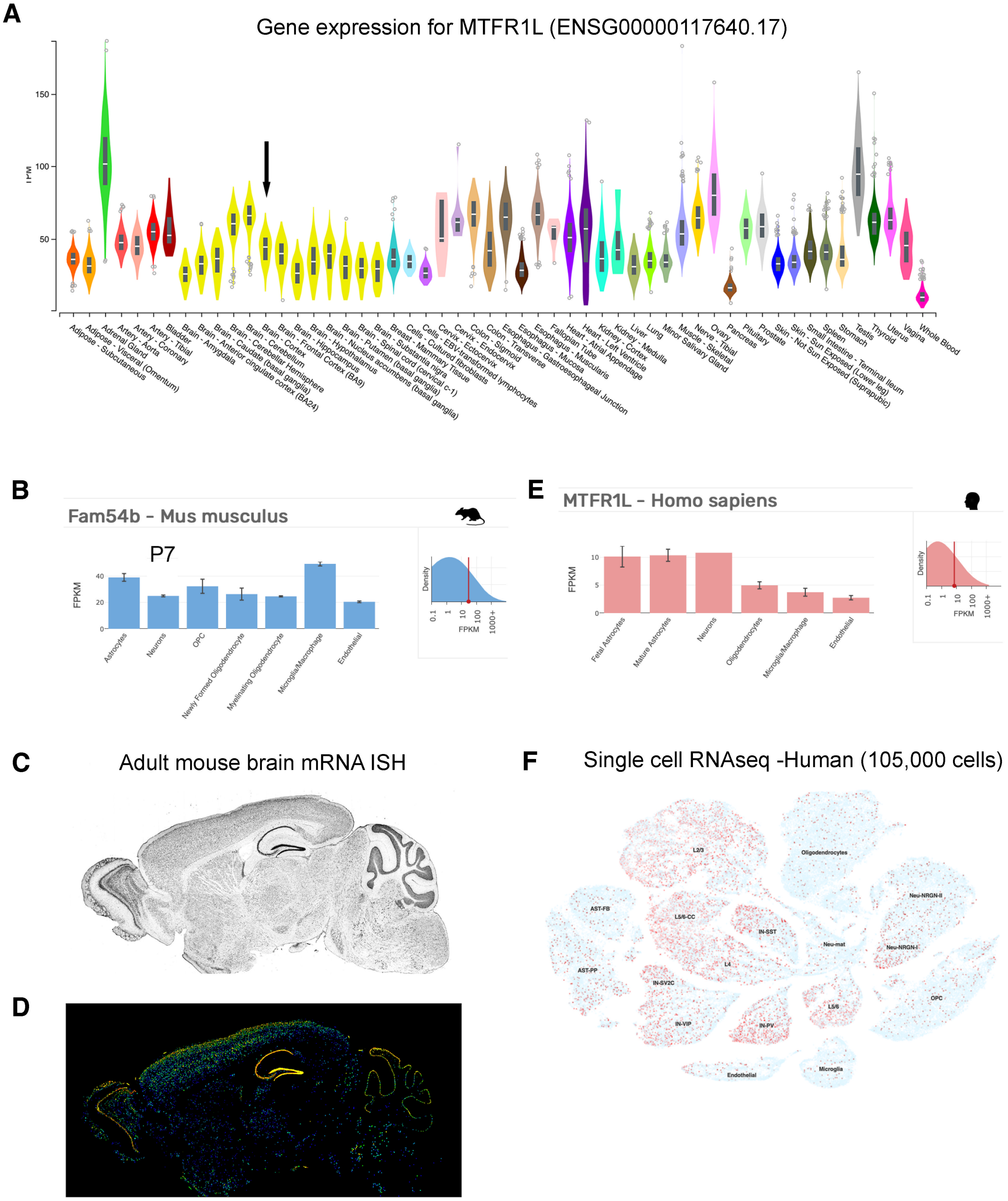

Figure S12, related to Figure 6. Analysis of MTFR1L expression in the brain.

(A) Publicly available RNAseq analysis showing gene expression of *MTFR1L* across human tissues. Arrow indicates expression in the cortex.

(B, C) Publicly available RNA-Seq data analysis of (B) *MTFR1L* homolog, *Fam54b*, expression in P7 mice from different cell types in the brain, and (C) *MTFR1L* expression in different cell types in the human brain.

(D, E) Publicly available (D) *in situ* hybridization of MTFR1L mRNA, and (E) MTFR1L protein expression, in adult mouse brain.

(F) Publicly available single cell RNAseq from human cortex showing expression of MTFR1L in various types of neurons including layer 2/3 pyramidal neurons.

Supplementary Figure S13

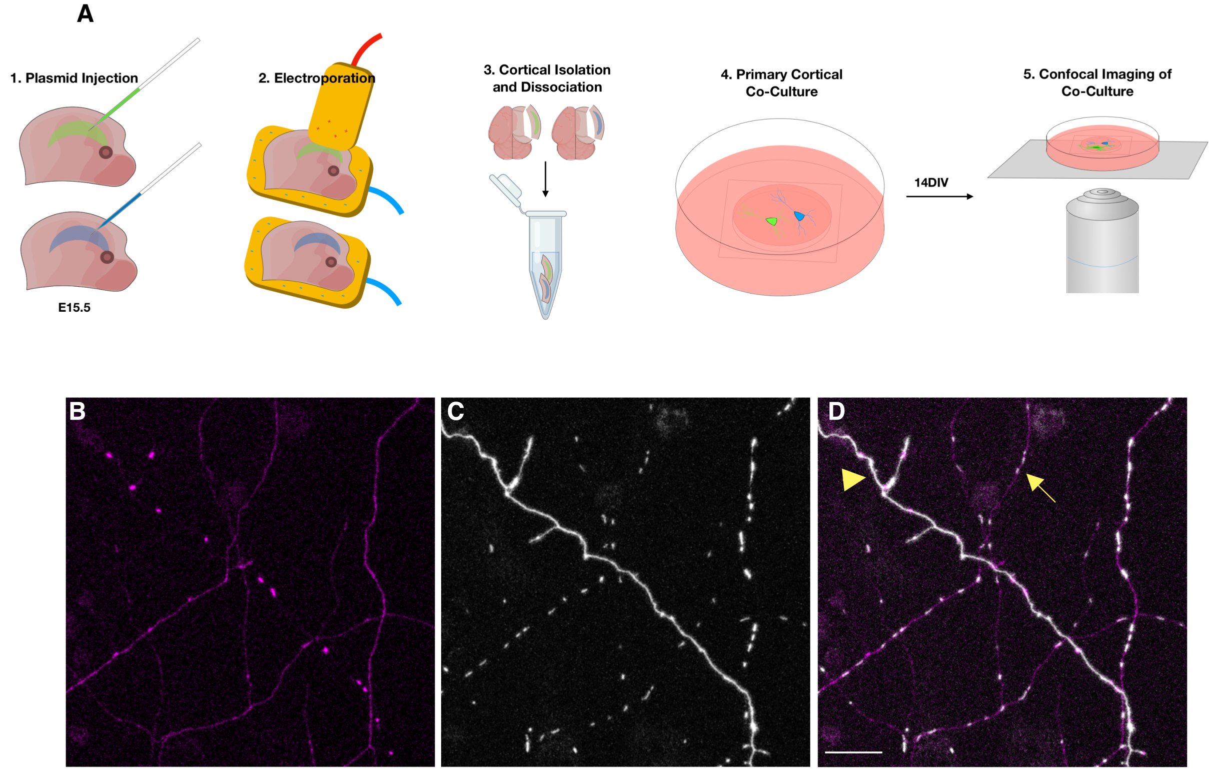

Figure S13, related to Figure 6. Technical description of the neuronal co-culture paradigm. Neuronal co-cultures were used to minimize introduction of confounding variables caused by plate-to-plate variability.

(A) Schematic depicting the co-culture technique used in the study (Figure 6A-H). Individual embryos were electroporated at E15.5 with an electroporation mixture for a single condition. A single cortical hemisphere from each condition was then dissected out, dissociated together, and plated. At DIV14, cells were fixed and imaged.

(B-D) Representative high magnification confocal image of axons from two conditions plated together with (B) showing axon from short-hairpin (sh)MTFR1L condition visualized with cytosolic Venus and mitochondria from sh non targeted (NT) condition visualized with mtYFP, (C) showing mitochondria from shMTFR1L condition visualized with mtDsRed and axon from shNT condition visualized with cytosolic tdTomato, and (D) showing the merge composite of (B)

and (C). Small arrow in (D) indicates shNT condition and plain bigger arrow represents shMTFR1L condition. Scale bar, 20 μm.

Table S1: List of primers

Primer name	Sequence 5'-3'
Site directed mutagenesis_S103D_Fwd	catgcagcgcaatgccgatgttcccaacctgcgt
Site directed mutagenesis_S103D_Rev	acgcaggttgggaacatcggcattgcgctgcatg
Site directed mutagenesis _S238D_Fwd	tctttgtccaaggccagcgactttgcagacatgatggg
Site directed mutagenesis _S238D_Rev	cccatcatgtctgcaaagtcgctggccttggacaaaga
Site directed mutagenesis _S103A_Fwd	tgcagcgcaatgccgctgttcccaacctg
Site directed mutagenesis _S103A_Rev	caggttgggaacagcggcattgcgctgca
Site directed mutagenesis _S238A_Fwd	ccatcatgtctgcaaaggcgctggccttggacaaag
Site directed mutagenesis _S238A_Rev	ctttgtccaaggccagcgcctttgcagacatgatgg
P2A_ primer_Fwd	atatgaattcatgtcaggaatggaagccactgtgacc
P2A _primer_Rev	atataccggtgaatttccttttctactgatatcttcttcctttagagcaaacttcctc
MTFR1L$^{\Delta 1\text{-}28}$_Infusion Fwd	ctcaagcttcgaattcatgggcaccaacctccccctt
MTFR1L$^{\Delta 1\text{-}28}$_Infusion_Rev	acccgatcccaccggtgaatttccttt
qPCR_GAPDH_Fwd	ggtgaaggtcggagtcaacg
qPCR_GAPDH_Rev	gagggatctcgctcctggaag
qPCR_OPA1_Fwd	ggctcctgacacaaaggaaa
qPCR_OPA1_Rev	tccttccatgagggtccatt
mCherry_Infusion_Fwd	tagcctcgaggtttaaacatggtgagcaagggcgag
mCherry_Infusion_Rev	gcggaattccggatccttacttgtacagctcgtccatgcc
MTFR1L_mCherry_Infusion_Fwd	ctagcctcgaggtttaaacatgtcaggaatggaagccac
MTFR1L no tag_Fwd	atatgaattcatgtcaggaatggaagccactgtgacc
MTFR1L no tag_Rev	atatctcgagtcaatttccttttctactgatatcttcttcctttagagcaaacttcctc
Primer S103_Fwd	atgcagcgcaatgccgctgttcccaacctg
Primer 103_Rev	caggttgggaacagcggcattgcgctgcat
Primer S238_Fwd	gtccaaggccagcgcctttgcagacatgatgg
Primer S238_Rev	ccatcatgtctgcaaaggcgctggccttggac
MTFR1L_FLAG_Fwd	ctgccggaattcaccatggattacaaggatgacgatgacaagtcaggaatggaagccactgtg
MTFR1L_FLAG_Rev	cgtcgcctcgagtcaatttccttttctactgatatc
